# Factors related to adverse long-term outcomes after mild traumatic brain injury in children: a scoping review

**DOI:** 10.1136/archdischild-2022-325202

**Published:** 2023-03-31

**Authors:** Sharea Ijaz, Lauren Scott, Sarah Dawson, Rebecca Wilson, Joni Jackson, Kate Birnie, Maria Theresa Redaniel, Jelena Savović, Ingram Wright, Mark D Lyttle, Julie Mytton

**Affiliations:** 1 NIHR ARC West, Population Health Sciences, University of Bristol, Bristol, UK; 2 Population Health Sciences, University of Bristol, Bristol, UK; 3 School of Psychological Science, University of Bristol, Bristol, UK; 4 Emergency Department, Bristol Royal Hospital for Children, Bristol, UK; 5 Research in Emergency Care Avon Collaborative Hub (REACH), University of the West of England, Bristol, Avon, UK; 6 School of Health and Social Wellbeing, University of the West of England, Bristol, UK

**Keywords:** adolescent health, child health, syndrome

## Abstract

**Objective:**

To identify demographic, premorbid and injury-related factors, or biomarkers associated with long-term (≥3 months) adverse outcomes in children after mild traumatic brain injury (mTBI).

**Design:**

Scoping review of literature.

**Patients:**

Children and adolescents with mTBI.

**Risk factors:**

Any demographic, premorbid and injury-related factors, or biomarkers were included. We excluded genetic and treatment-related factors.

**Main outcome measures:**

Postconcussion syndrome (PCS), recovery.

**Results:**

Seventy-three publications were included, reporting 12 long-term adverse outcomes, including PCS in 12 studies and recovery in 29 studies. Additional outcomes studied were symptom scores/severity (n=22), quality of life (n=9) and cognitive function (n=9). Forty-nine risk factors were identified across studies. Risk factors most often assessed were sex (n=28), followed by age (n=23), injury mechanism = (n=22) and prior mTBI (n=18). The influence of these and other risk factors on outcomes of mTBI were inconsistent across the reviewed literature.

**Conclusions:**

The most researched risk factors are sex, age and mechanism of injury, but their effects have been estimated inconsistently and did not show a clear pattern. The most studied outcomes are recovery patterns and symptom severity. However, these may not be the most important outcomes for clinicians and patients. Future primary studies in this area should focus on patient-important outcomes. Population-based prospective studies are needed that address prespecified hypotheses on the relationship of risk factors with given outcomes to enable reliable prediction of long-term adverse outcomes for childhood mTBI.

**WHAT IS ALREADY KNOWN ON THIS TOPIC**

- Mild traumatic brain injury (mTBI) is common in childhood and can lead to prolonged symptoms in about one-third of children.
- Persisting symptoms can hinder development and reduce quality of life.
- There is a dearth of predictive tools and limited evidence on factors placing a child at risk of developing prolonged symptoms.

**WHAT THIS STUDY ADDS**

- This study collated potential demographic, premorbid and injury-related factors, or biomarkers for long-term adverse outcomes of childhood mTBI.
- Forty-nine risk factors for 12 different outcomes were identified across 73 studies.
- The associations between any of the risk factors and adverse outcomes were not consistent.

**HOW THIS STUDY MIGHT AFFECT RESEARCH, PRACTICE OR POLICY**

- The inconsistency of findings indicates a need for consensus on definitions underpinning measurement.
- Robust primary studies that follow-up children with mTBI long-term are required to reliably predict adverse outcomes.

## Introduction

Almost half a million children attend hospital annually after a traumatic brain injury (TBI) in the UK.^1^ More than 90% are mild,^2^ although the definition of ‘mild’ varies, and there is no consensus on severity thresholds based on symptoms and signs.^3^ Symptoms of mild TBI (mTBI) can persist for several months (postconcussion syndrome (PCS))^4^ in up to one-third of patients. PCS lacks a widely accepted definition, with different criteria used for diagnosis (Diagnostic and Statistical Manual of Mental Disorders, fourth edition and International Classification of Diseases 10).^5^ It presents with subjective physical complaints (eg, headache, dizziness), and cognitive, emotional and behavioural changes. These symptoms can appear early or late after the injury, may become chronic or permanent and negatively impact development and quality of life for affected children.^6^

**Figure 1:**
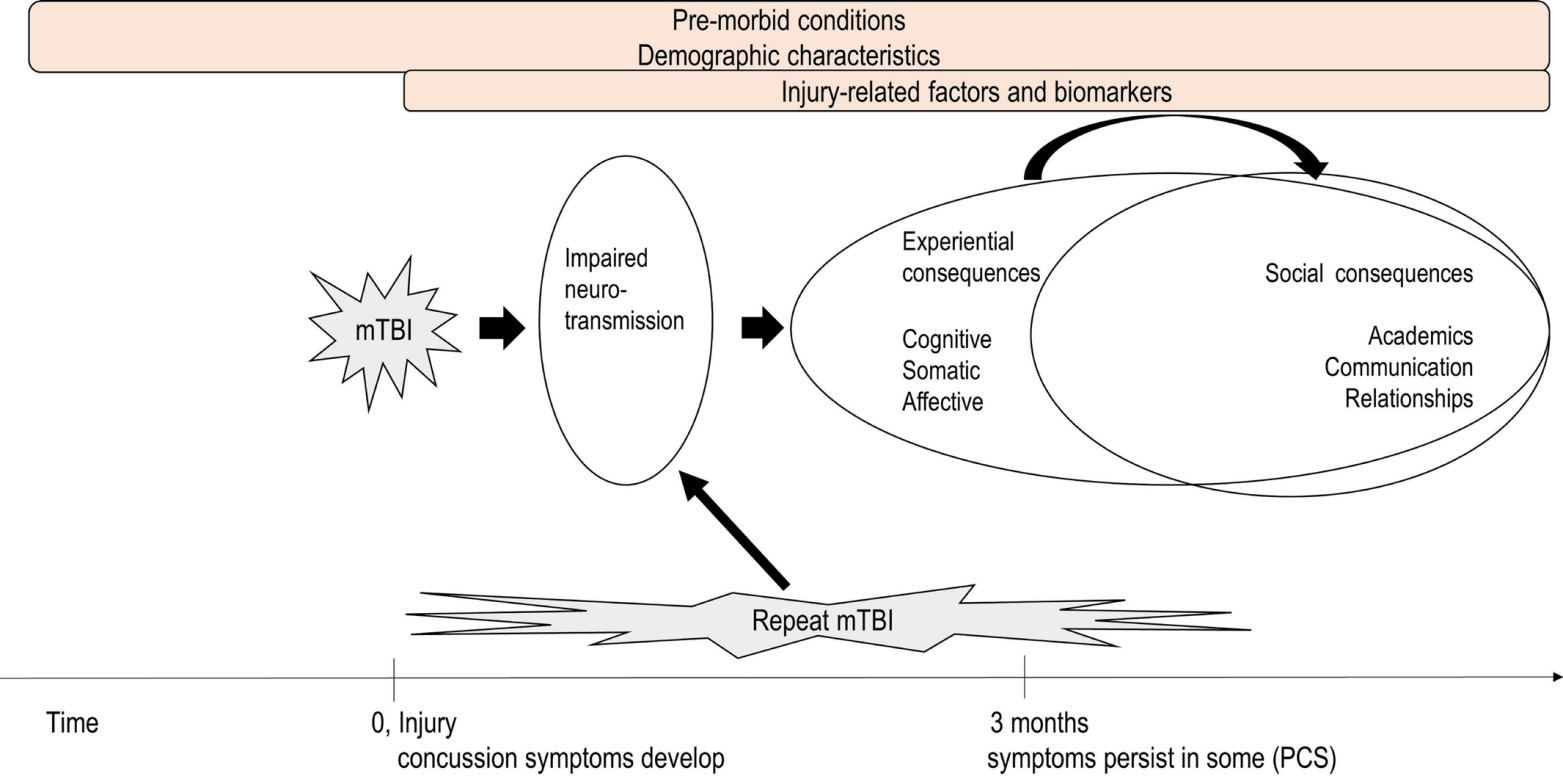
Mild traumatic brain injury (mTBI) and how potential factors may affect symptoms after mTBI. PCS, postconcussion syndrome.

Research on mTBI pathophysiology has explored links between symptoms and potential individual or environmental factors.^4 7 8^ mTBI can result in disturbed neurotransmission which, when combined with personal or environmental characteristics, can lead to different types and durations of symptoms post injury (figure 1).

There is no widely accepted, validated prediction tool to identify children at risk of developing long-term sequelae of mTBI. Limited evidence on syndrome characterisation precludes developing interventions for PCS.^9^ Knowing who to target would help develop and refine interventions to prevent or treat symptoms and lower morbidity.

In this review, we aimed to identify factors associated with long-term adverse outcomes of mTBI from published research and identify key areas for future research.

### Objective

To identify from the literature the demographic, premorbid and injury-related factors, or biomarkers associated with long-term (≥3 months) adverse outcomes in children after mTBI.

## Methods

We followed accepted methods for scoping reviews and used an iterative process to define research questions, extract data and summarise results.^10–12^ We report the review as per accepted guidelines.^13^

We included observational studies reporting any demographic, premorbid and injury-related factors, or biomarker risk factors for adverse outcomes 3 months or longer after mTBI in children under 18 years. We searched three electronic databases and references lists of included studies and published systematic reviews to identify peer reviewed and grey literature in January 2022. Screening of titles and abstracts and full texts was done in duplicate. Data extraction was done by one author, and the data items included study design, author, location, year of publication, participants, risk factors identified or studied and outcome(s). We did not extract effect estimates and did not perform risk of bias assessments. Findings were summarised into a table of risk factors studied for each outcome and in figures presenting long-term adverse outcomes and risk factors studied in literature respectively. A stakeholder consultation helped refine the synthesis and findings. Detailed methods are available in online supplemental file.

10.1136/archdischild-2022-325202.supp1 Supplementary data

## Results

### Search results and selection

Of 8039 unique references, 678 underwent full-text assessment, and 73 publications were included (see online supplemental table of characteristics of included studies). Most (n=42) originated from the USA, followed by Canada (n=14), Europe (n=6) and Australia (n=5), and most were from secondary care settings (n=50). The median sample size was 146 participants (IQR 72–285). Publication dates ranged from 1999 to 2022, with the latest cohort enrolled between 2015 and 2021.

Male participants were more common (>50% males) in 46 studies. Median age was 13.6 years (IQR 12–15) across the 64% of included studies reporting mean age. Half the studies reported race, in which participants were mostly white (median 75%; IQR 68%–84%).

### Outcomes assessed

Our primary outcome PCS was reported in 12 studies only, though descriptions and definitions of PCS differed (table 1).

**Table 1:** Definitions of postconcussion syndrome used across included studies reporting this outcome

Study ID: author date	PCS definition and measurement reported
Babcock 2013	Diagnostic and Statistical Manual of Mental Disorders, fourth edition (DSMIV): presence of three or more symptoms on the Rivermead Post Concussion Symptoms Questionnaire (RPQ) that were rated as worse (score of >2) than before mTBI at 3 months
Chendrasekar 2020^36^	Author defined: questionnaire assessing presence of any concussion symptoms; measured 4–68 months after mTBI
Corwin 2020	International Classification of Diseases (ICD) 10 definition: symptoms that persisted beyond 28 days following mTBI; measured at 28 days and 3 months
Durish 2019	ICD 10 definition; presence of one or more symptoms reported to be associated with the concussion that persist for longer than 1 month post-mTBI; measured at 28 days and 3 months
Gravel 2020	ICD 10 definition: an increase of at least three symptoms on the child Post-Concussion Symptom Inventory as compared with the child’s baseline behaviour prior to mTBI at 1 week; measured at 1, 4 and 12 weeks
Haase 2015	DSMIV definition: presence of three to six symptom categories occurring within 3 months post-mTBI and evidence of neuropsychological dysfunction
Howell 2018^14^	ICD definition: symptoms lasting more than 28 days; measured at 3 months post-mTBI
Jeckell 2019^37^	DSMIV definition: presence of 3 or more symptoms that lead to impairment in functioning and last no less than 3 months post-mTBI
Kelmendi 2021	DSMIV definition: the presence of 3 or more symptoms on the RPQ scaler that rated as worse (score of ≥2) than at preinjury, at 3 months post-mTBI
Olsson 2013	DSMIV definition: a measure of PCS was derived from parent’s responses on the Child Behaviour Checklist (CBCL), by scoring only the items on CBCL that matched on face validity to one of the Post-Concussion Disorder (PCD) symptoms in criterion C of PCD research criteria in DSMIV
Preiss-Farzanegan 2009	DSMIV definition: the presence, nature and extent of postconcussive symptoms on RPQ scale, administered over the telephone 3 months post-mTBI
Riemann 2021	ICD 10 definition; at least 3 of the following symptoms: headaches, dizziness, sleep disturbance, fatigue, being irritable/easily angered, forgetfulness/poor memory and poor concentration on RPQ scale (severity rating score ≥2); measured at 3 and 6 months

mTBI, mild traumatic brain injury; PCS, postconcussion syndrome.

**Figure 2:**
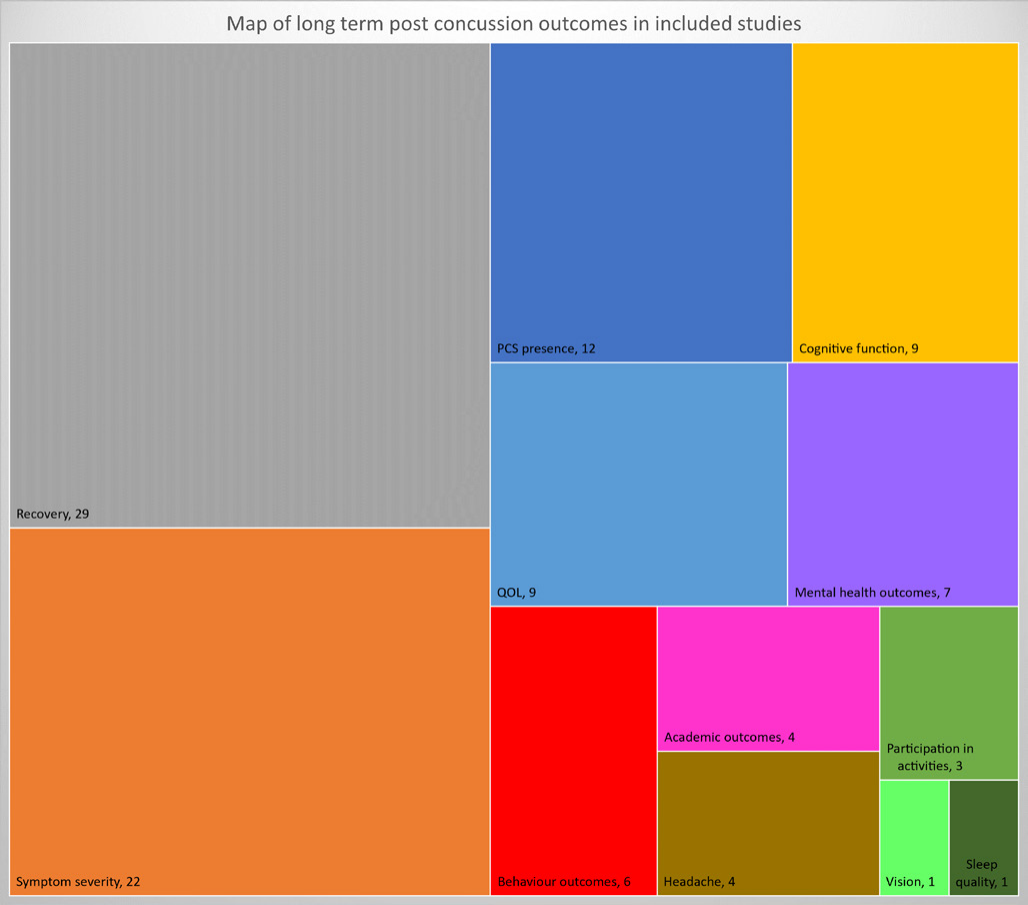
Map of long-term outcomes of childhood mTBI in literature. The size of the block indicates the relative size of literature on the outcome, with numbers of studies reporting each. mTBI, mild traumatic brain injury.

Symptom severity or scores (rather than presence of a set of symptoms) were reported in 22 studies, using various scales. Recovery was reported in 29 studies. Other outcomes were reported less frequently (figure 2).

Follow-up schedules varied, with 3-month outcomes reported most often (n=43); the longest follow-up was 7 years.

### Risk factors

**Figure 3:**
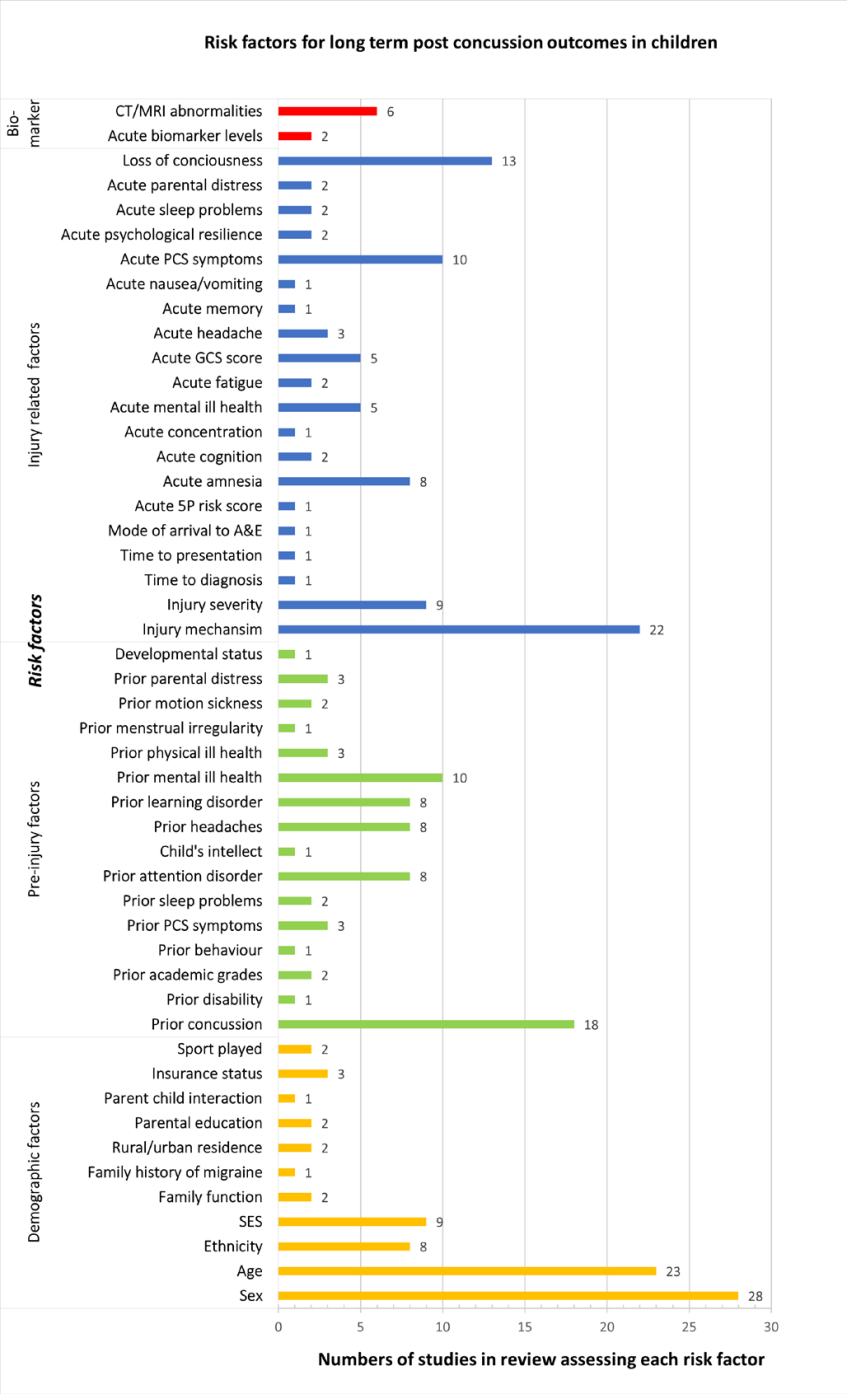
Risk factors for long-term outcomes of childhood mTBI in literature. mTBI, mild traumatic brain injury; PCS, postconcussion syndrome; 5P risk score.Predicting and Preventing Post-concussive Problems in Pediatrics risk score; SES, Socioeconomic status; A&E, Accident and Emergnecy; GCS, Glasgow coma scale; CT/MRI, Computerised tomography/Magnetic resonance imaging.

Forty-nine risk factors were reported across 73 studies, of which sex (28; 37%), age (23; 31%) and injury mechanism (22; 30%) were most frequently assessed. Fourteen factors were assessed in single studies only (figure 3).

### Relationship of outcomes with risk factors

#### Postconcussion syndrome

Twelve studies assessed effect of risk factors on PCS occurrence. Evidence was inconsistent for age and sex, assessed in three and four studies, respectively. Premorbid anxiety and depression were assessed in two studies each and were unrelated to PCS in both. All other factors were assessed in single studies (see online supplemental table of risk factors).

#### Severity of symptoms

Twenty-two studies reported symptom severity scores. Evidence was inconsistent for sex, age, parental education, SES, prior mTBI, prior mental ill health, mechanism of injury and ADHD. Immediate postinjury sleep problems, low resilience, high PTSD symptoms and high serum S100B levels within 6 hours of injury in single studies were reported as related to greater PCS symptoms. Other factors were unrelated to symptom severity ((see online supplemental table of risk factors).

#### Recovery

Twenty-nine studies reported recovery trajectory (online supplemental table of risk factors), exploring factors including prior mTBIs, injury mechanism, age, sex, premorbid headaches, prior learning difficulties and loss of consciousness. Studies reporting poor recovery found it to be predicted by high postinjury PCS scores (n=2) and postinjury parental distress (n=3). Single studies assessing delayed recovery found this was predicted by higher composite post-mTBI risk scores (5P score),^14^ delayed diagnosis, low resilience, higher depression and internalising symptoms, amnesia, continued activity participation, injury severity and acute post-mTBI fatigue.

#### Cognitive function

Nine studies reported cognitive outcomes. Evidence was inconsistent on age, sex, SES, ethnicity and MRI findings. Rural residence, preinjury attention deficit hyperactivity disorder (ADHD), prior cognitive problems, absence of headache at presentation and higher serum S100B after mTBI were linked to poor cognitive outcome in single studies (online supplemental table of risk factors).

#### Quality of life (QOL)

Nine studies reported QoL. High immediate post-mTBI PCS symptoms, prior parental distress and poor parent child interactions were associated with poor QoL outcomes. Evidence was inconsistent on age, sex, SES and injury mechanism.

#### Mental health outcomes

Poor mental health outcomes, reported by seven studies, were predicted by high immediate post-mTBI PCS symptoms in two studies and by premorbid anxiety/depression, low SES, low resilience, and weaker connectivity on MRI in single studies. There was inconsistent evidence on effect of sex and prior mTBI (online supplemental table of risk factors).

All other outcomes and associated risk factors are listed in online supplemental table of risk factors.

#### Risk factors not assessed for any outcome

Predefined risk factors that we did not identify in the literature for any outcome were parental drug or alcohol abuse, family size or social services involvement.

## Discussion

We identified 49 risk factors across 73 publications, of which 14 were only assessed in single studies. Four factors, preidentified as potentially relevant, were not evaluated in any studies.

While sex and age were reported in more than 20 studies each, findings across studies were disparate. Overall, girls seemed to have poorer outcomes, similar to recent evidence indicating girls are more likely to suffer symptoms after mTBI and recover later.^15^ Results for age were more varied: four studies reported either older or younger age as a risk factor for poorer outcomes, while five found age was not associated with outcomes. Different cut-offs for age groups (eg, <10 vs ≥10 years; <13 vs ≥13 years) have been tested in primary studies, creating difficulties in evaluating the influence of age on consequences of mTBI across studies. To enable reliable predictions of outcomes by age we need large follow-up studies with a priori hypotheses and agreement across researchers for appropriate age cut-offs.

Data on some demographic factors such as SES, family function and ethnicity were sparse, and findings were inconsistent. Since these wider determinants are known to modify many health outcomes and behaviours,^16–18^ these should be better examined in future studies, for example, by defining these a priori, measuring at baseline using valid scales and testing their causal relationship with different adverse outcomes. Including these measures as a minimum data set in future studies (or routine data collection at healthcare interaction) would amplify potential for meta-analyses of future work, even if individual studies are not sufficiently powered.

The next most commonly studied factor was mechanism of injury (n=22). There were inconsistent effects from different mechanisms of mTBI, although road traffic injuries were found by some to be predictive of poorer outcome. Prior mTBI was unrelated to poor outcomes in 12 out of 18 studies assessing this, and the only outcome it consistently predicted was persistent headaches (n=3).

Evidence on all other risk factors was also inconsistent; presence or absence of these factors did not always predict similar outcome patterns across studies. Several injury-related factors are easily measurable at presentation postinjury. However, measurement is often variable, for example, proxy versus self-rated symptoms versus clinician assessment, which may explain inconsistent findings.

Blood biomarkers have been increasingly suggested as potential predictors for recovery from mTBI.^19^ However, this is based on animal or in vitro models. Restricted to human studies, evidence on biomarkers was scarce (n=2) with higher levels of S100B postinjury related to poor outcomes. Several others (eg, IL6, glial fibrillar acidic protein; ubiquitin C-terminal hydrolase-L1; myelin basic protein; neuron-specific enolase; and prostaglandin D synthase) can be measured using blood tests at presentation. A recent evidence review to inform guidance on prognosticating long-term adversity using blood and MRI found limited evidence,^20^ with a consequent recommendation that biomarkers be researched as potential indicators of later outcomes in robust studies.^20 21^ An ongoing study is assessing their prognostic potential in delayed recovery from mTBI in children presenting at hospital emergency departments.^22^ Similar studies in other settings and populations for important outcomes are needed to fill the gaps in the evidence base.

Most studies relied on retrospective accounts of preinjury difficulties, which have inherent potential for recall bias. From included studies and stakeholder consultations, it seems symptoms commonly reported after mTBI (sleep/concentration problems, headaches) may already be present prior to injury. Baseline (preseason) testing, as undertaken in some sports in regions such as the USA, allows assessment of change in symptoms on comparable measures and recording of any existing risk factors. Such prospective surveillance (measuring demographic and potential risk factors as well as current existence of any symptoms such as headaches that can be considered a post mTBI outcome) in school level sports and its use in future research can improve reliability of post mTBI findings as new or worse symptoms, facilitating reliable relating of postinjury findings to baseline risk factors. Healthcare services in many settings do not currently follow-up children long-term post-mTBI; were this to happen, the understanding of the natural progression of outcomes following mTBI would be greatly enriched. These measurements could be part of a core outcome set for implementation in the UK and other healthcare systems – either collated by the clinician in Electronic Health Records or by patients/families in a mobile device application (as was done during COVID).

### Comparison with other reviews

We found 15 systematic reviews of one or more risk factors for various mTBI outcomes.^20–34^ Eligibility criteria for these reviews varied and follow-up was not well defined, leading to diverse findings. For example, two reported older children had poorer outcomes^15 16^; however, these included different age ranges (>20 years vs 2–18 years), outcomes (PCS vs quality of life) and methods, so comparisons are more nuanced. All reviews found the quality low and data sparse for any risk factor and outcome relationship, and these also recommended large representative cohort studies to establish risk factors. Because some factors (eg, known to social services, benefit receipt, social class) have different meanings across the world, we consider it important that country or region-specific cohort studies are conducted to establish the value of risk factors for specific outcomes. As most children recover within 3 months, future studies to identify children less likely to recover should have longer follow-up periods after mTBI. To this end, our review’s findings will inform a study using UK primary and secondary care records.^35^

This scoping review explored evidence reporting associations of demographic, premorbid and injury-related factors with adverse outcomes following mTBI and whether biomarkers are potential predictors. The inconsistency of findings indicates a need for consensus on definitions underpinning measurement of potential risk factors and outcomes and for studies specifying a priori hypotheses and including baseline measures where feasible. The role of biomarkers appears under-researched as does our understanding of behavioural responses of children, their parents and healthcare providers to post-mTBI symptoms, which may improve our understanding of why such variation has been found to date.

### Limitations

This was not a systematic review. As such, we did not perform study bias assessments or pooling of effects across studies. Our aim was to scope existing literature for all factors studied to date as potential risk factors for specific outcomes. While we identified 49 potential factors, the evidence is limited for any of these, and several prespecified potentially relevant factors are missing from existing research. We included all ‘author-defined mTBI’ to scope the full breadth of the evidence, but a disadvantage of this is substantial heterogeneity across studies (eg, different inclusion thresholds for CT abnormalities). We did not address moderate or severe TBI as these often require active management, whereas mTBI is expected to resolve spontaneously. mTBI is especially important to study as this is the severity ascribed to most children, and identifying those who will experience long-term symptoms is challenging for clinicians.

## Conclusion

In the substantial research on risk factors associated with poor long-term outcomes of mTBI, the three most researched are sex, age and mechanism of injury. However, their effects have been measured variably and do not show a clear pattern. The most commonly reported outcomes of recovery and symptom severity may not be the most important outcomes for clinicians and patients, and a collaborative core outcome set should be developed. Future studies should correlate risk factors with prioritised outcomes in high-quality primary observational studies with follow-up of several months duration. True quantification of the impact of mTBI in childhood will only be possible if preinjury data are routinely collected, either by healthcare systems, organised sports or self-report. Research is also needed to clarify the severity of some risk factor categories, such as some age groups or some injury mechanisms for relationship with worse outcomes.

## Data Availability

All data relevant to the study are included in the article or uploaded as supplementary information.
