# Supplementary material for: Factors related to adverse long-term outcomes after mild traumatic brain injury in children: a scoping review
Source: Arch Dis Child. 2023 Mar 31;108(6):492–7. doi: 10.1136/archdischild-2022-325202 (PMC10314050; doi:10.1136/archdischild-2022-325202)
Supplement: Supplementary data [file archdischild-2022-325202supp001.pdf]

Appendices

Methods:

Search

We searched electronic databases (Medline, EMBASE, CINAHL) for peer reviewed and grey literature (conference abstracts and theses) in January 2022 (see below). We searched reference lists to identify additional relevant studies.

Search Strategies

Ovid MEDLINE(R) ALL <1946 to January 17, 2022>

1 Post-Concussion Syndrome/ 1396
2 ((post or persistent or unresolved or delayed) adj4 concuss*).mp. or postconcuss$.tw,kf. 3912
3 (commotio cerebri or post trauma* encephalopath* or posttrauma* encephalopath*).mp. or ((post commotion or postcommotion or post contusi* or postcontusi* or post head injur*) adj2 syndrom*).tw,kf. or (post trauma* syndrom* or posttrauma* syndrom*).tw,kf. or shell shock*.tw,kf. 540
4 Brain Concussion/ or Brain Injury, Chronic/ 10688
5 ((post or persistent or unresolved or delayed) adj4 (brain or skull or head or injur*)).mp. 28625
6 4 and 5 872
7 *Brain Concussion/co 1074
8 following concuss*.mp. 500
9 or/1-3,6-8 5805
10 adolescent/ or child/ or child, preschool/ or infant/ 3465219
11 adolescent.hw. 2152979
12 (adolescent or child).hw. 3269095
13 (infant? or toddler* or child* or boy* or girl* or infant* or juvenil* or minors or paediatric* or pediatric* or school* or preschool or pre-school* or kids or kindergarten or nursery or adolesc* or preadolesc* or pre-adolesc* or pubert* or pubescen* or prepube* or pre-pube* or high-school or highschool* or teen* or young or youth* or student* or undergrad* or college or campus).ti,ab,kf. 3202326
14 (infant* or child* or adolesc*).jw. 207167
15 or/10-14 4946317
16 9 and 15 2497
17 ((infant? or toddler* or child* or boy* or girl* or infant* or juvenil* or minors or paediatric* or pediatric* or school* or preschool or pre-school* or kindergarten or nursery or adolesc* or preadolesc* or pre-adolesc* or pubert* or pubescen* or prepube* or pre-pube* or high-school or highschool* or teen* or young or youth* or student* or undergrad*) adj3 concuss*).ti,kf. 814
18 16 or 17 2942
19 (2020* or 2021* or 2022*).yr,dp,dt,ep,ez. 3331644
20 18 and 19 669

****

The Cochrane Library, Issue 12 of 12, 2022

#1 MeSH descriptor: [Post-Concussion Syndrome] this term only 162

#2 (((post or persistent or unresolved or delayed) NEAR/4 concuss*). or postconcuss*):ti,ab,kw 502

#3 (“commotio cerebri” or (post next trauma* next encephalopath*) or (posttrauma* next encephalopath*) or (post next trauma* next syndrom*) or (posttrauma* next syndrom*) or (shell next shock*)):ti,ab,kw 23
#4 ((post next commotion) or postcommotion or (post next contusi*) or postcontusi* or (post next head next injur*)):ti,ab,kw 15
#5 ("brain concussion" or "brain injury"):kw 2869
#6 ((post or persistent or unresolved or delayed) NEAR (brain or skull or head or injur*)):ti,ab,kw 3003
#7 (#5 and #6) 366
#8 MeSH descriptor: [Brain Concussion] explode all trees and with qualifier(s): [complications - CO] 56
#9 ((following or after) near/2 concuss*):ti,ab,kw 143
#10 (#1 OR #2 OR #3 OR #4 OR #7 OR #8 OR #9) 867
#11 (infant* or toddler* or child* or boy* or girl* or infant* or juvenil* or minors or paediatric* or pediatric* or school* or preschool or pre-school* or kids or kindergarten or nursery or adolesc* or preadolesc* or pre-adolesc* or pubert* or pubescen* or prepube* or pre-pube* or high-school or highschool* or teen* or young or youth* or student* or undergrad* or college or campus):ti,ab,kw 412257
#12 (#10 and #11) 389
#13 ((infant* or toddler* or child* or boy* or girl* or infant* or juvenil* or minors or paediatric* or pediatric* or school* or preschool or pre-school* or kids or kindergarten or nursery or adolesc* or preadolesc* or pre-adolesc* or pubert* or pubescen* or prepube* or pre-pube* or high-school or highschool* or teen* or young or youth* or student* or undergrad* or college or campus or sport*) NEAR concuss*):ti,ab,kw 302
#14 (sport* next related concussion*):ti,ab,kw 118
#15 (#12 OR #13 OR #14) 544 Trials
Date limited: 01/03/2020 to 17/01/2022 n=159

****

Ovid Embase <1974 to 2022 January 14>
1 postconcussion syndrome/ 2675
2 ((post or persistent or unresolved or delayed) adj4 concuss*).mp. or (postconcuss* or post concuss*).tw,kw. 5203
3 (commotio cerebri or post trauma* encephalopath* or posttrauma* encephalopath*).mp. or ((post commotion or postcommotion or post contusi* or postcontusi* or post head injur*) adj2 syndrom*).tw,kw. or (post trauma* syndrom* or posttrauma* syndrom*).tw,kw. or shell shock*.tw,kw. 660
4 exp concussion/ 13769
5 ((post or persistent or unresolved or delayed) adj4 (brain or skull or head or injur*)).mp. 44737
6 4 and 5 1182
7 exp concussion/ and complication.fs. 1032
8 (following adj2 concuss*).mp. 1274
9 or/1-3,6-8 8195
10 (infant* or adolescent or child* or pediatric* or paediatric*).jw,hw. 3790369
11 (infant? or toddler* or child* or boy* or girl* or infant* or juvenil* or minors or paediatric* or pediatric* or school* or preschool or pre-school* or kids or kindergarten or nursery or adolesc* or preadolesc* or pre-adolesc* or pubert* or pubescen* or prepube* or pre-pube* or high-school or highschool* or teen* or young or youth* or student* or undergrad* or college or campus).ti,ab,kw. 3988204
12 10 or 11 5361688
13 ((infant? or toddler* or child* or boy* or girl* or infant* or juvenil* or minors or paediatric* or pediatric* or school* or preschool or pre-school* or kindergarten or nursery or adolesc* or preadolesc* or pre-adolesc* or pubert* or pubescen* or prepube* or pre-pube* or high-school or

highschool* or teen* or young or youth* or student* or undergrad*) adj3 concuss*).ti,kw. 1570
14 9 and 12 3328
15 13 or 14 4140
16 limit 15 to conference abstract status 1069
17 15 not 16 3071
18 (2020* or 2021* or 2022*).yr,dc,dp. 4212248
19 17 and 18 790

****
APA PsycInfo <1806 to January Week 2 2022>
1 Post-Concussion Syndrome.mp. 591
2 (((post or persistent or unresolved or delayed) adj4 concuss*) or postconcuss*).tw,id. 2085
3 (commotio cerebri or post trauma* encephalopath* or posttrauma* encephalopath* or ((post commotion or postcommotion or post contusi* or postcontusi* or post head injur*) adj2 syndrom*) or (post trauma* syndrom* or posttrauma* syndrom*) or shell shock*).tw,id. 431
4 (exp Brain Concussion/ or exp brain injuries/) and ((post or persistent or unresolved or delayed) adj4 (brain or skull or head or injur*)).ti,ab,id. 3010
5 following concuss*.tw,id. 225
6 (sport? adj2 concuss*).tw,id. 1048
7 or/1-6 5845
8 (adolescent or child).hw. 231814
9 (infant? or toddler* or child* or boy* or girl* or infant* or juvenil* or minors or paediatric* or pediatric* or school* or preschool or pre-school* or kids or kindergarten or nursery or adolesc* or preadolesc* or pre-adolesc* or pubert* or pubescen* or prepube* or pre-pube* or high-school or highschool* or teen* or young or youth* or student* or undergrad* or college or campus).tw,id,hw. 1804219
10 (infant* or child* or adolesc*).jw. 163305
11 or/8-10 1814083
12 7 and 11 1759
13 (2020* or 2021* or 2022*).yr,an. 329254
14 12 and 13 329

****

Web of Science
Indexes=SCI-EXPANDED, SSCI Timespan=All years
Limit #3 (2020 to 2022) n=592
#3 [2944] (#1 or #2)
#2 [1118] TI=(((infant* or toddler* or child* or boy* or girl* or infant* or juvenil* or minors or paediatric* or pediatric* or school* or preschool or pre-school* or kindergarten or nursery or adolesc* or preadolesc* or pre-adolesc* or pubert* or pubescen* or prepube* or pre-pube* or high-school or highschool* or teen* or young or youth* or student* or undergrad*) NEAR/2 concuss*))
#1 [2224] TS=((((post or persistent or unresolved or delayed) NEAR concuss*) OR (postconcuss* or “post concuss*”) OR (“commotio cerebri” or “post trauma* encephalopath*” or “posttrauma* encephalopath*”) OR ((“post commotion” or postcommotion or “post contusi*” or postcontusi* or “post head injur*”) NEAR syndrom*) OR (“post trauma* syndrom*” or “posttrauma* syndrom*” OR “shell shock")) AND (infant* or toddler* or child* or boy* or girl* or infant* or juvenil* or minors or paediatric* or pediatric* or school* or preschool or pre-school* or kids or kindergarten or nursery or adolesc* or preadolesc* or pre-adolesc* or pubert* or pubescen* or prepube* or pre-pube* or high-school or highschool* or teen* or young or youth* or student* or undergrad* or college or campus))

Ijaz S, *et al. Arch Dis Child* 2023; 108:492–497. doi: 10.1136/archdischild-2022-325202

****

EBSCO ERIC (Education Resources Information Center), BEI (British Education Index)
TI ((TBI or mTBI or “brain injur*” or "head injur*" or concuss* or postconcuss* or “post concuss*” or “commotio cerebri” or “post trauma* encephalopath*” or “posttrauma* encephalopath*” or “post commotion” or postcommotion or “post contusi*” or postcontusi* or “post head injur*” or “post trauma* syndrom*” or “posttrauma* syndrom*” or “shell shock”)) OR AB ((TBI or mTBI or “brain injur*” or "head injur*" or concuss* or postconcuss* or “post concuss*” or “commotio cerebri” or “post trauma* encephalopath*” or “posttrauma* encephalopath*” or“post commotion” or postcommotion or “post contusi*” or postcontusi* or “post head injur*” or “post trauma* syndrom*” or “posttrauma* syndrom*” or “shell shock”)) OR SU ((“Head Injuries” or "Brain Damage" or "Brain-Damaged Children” or "Concussion Policies”)) 1615
Date limited: 2020-2022 n=64

****

Ovid MEDLINE(R) and Epub Ahead of Print, In-Process & Other Non-Indexed Citations and Daily <1946 to March 10, 2020>

----

1 Post-Concussion Syndrome/ (1055)
2 ((post or persistent or unresolved or delayed) adj4 concuss*).mp. or postconcuss$.tw,kf. (3176)
3 (commotio cerebri or post trauma* encephalopath* or posttrauma* encephalopath*).mp. or ((post commotion or postcommotion or post contusi* or postcontusi* or post head injur*) adj2 syndrom*).tw,kf. or (post trauma* syndrom* or posttrauma* syndrom*).tw,kf. or shell shock*.tw,kf. (507)
4 Brain Concussion/ or Brain Injury, Chronic/ (8570)
5 ((post or persistent or unresolved or delayed) adj4 (brain or skull or head or injur*)).mp. (24564)
6 4 and 5 (572)
7 *Brain Concussion/co (927)
8 following concuss*.mp. (375)
9 or/1-3,6-8 (4747)
10 adolescent/ or child/ or child, preschool/ or infant/ (3199361)
11 adolescent.hw. (1996477)
12 (adolescent or child).hw. (3018454)
13 (infant? or toddler* or child* or boy* or girl* or infant* or juvenil* or minors or paediatric* or pediatric* or school* or preschool or pre-school* or kids or kindergarten or nursery or adolesc* or preadolesc* or pre-adolesc* or pubert* or pubescen* or prepube* or pre-pube* or high-school or highschool* or teen* or young or youth* or student* or undergrad* or college or campus).ti,ab,kf. (2874210)
14 (infant* or child* or adolesc*).jw. (188291)
15 or/10-14 (4532950)
16 9 and 15 (1986)
17 ((infant? or toddler* or child* or boy* or girl* or infant* or juvenil* or minors or paediatric* or pediatric* or school* or preschool or pre-school* or kindergarten or nursery or adolesc* or preadolesc* or pre-adolesc* or pubert* or pubescen* or prepube* or pre-pube* or high-school or highschool* or teen* or young or youth* or student* or undergrad*) adj3 concuss*).ti,kf. (620)
18 16 or 17 (2344)

****

The Cochrane Library, Issue 3 of 12, 2020

#1 MeSH descriptor: [Post-Concussion Syndrome] this term only 125

#2 (((post or persistent or unresolved or delayed) NEAR/4 concuss*). or postconcuss*):ti,ab,kw 387
#3 (“commotio cerebri” or (post next trauma* next encephalopath*) or (posttrauma* next encephalopath*) or (post next trauma* next syndrom*) or (posttrauma* next syndrom*) or (shell next shock*)):ti,ab,kw 21
#4 ((post next commotion) or postcommotion or (post next contusi*) or postcontusi* or (post next head next injur*)):ti,ab,kw 15
#5 ("brain concussion" or "brain injury"):kw 2285
#6 ((post or persistent or unresolved or delayed) NEAR (brain or skull or head or injur*)):ti,ab,kw 2414
#7 (#5 and #6) 285
#8 MeSH descriptor: [Brain Concussion] explode all trees and with qualifier(s): [complications - CO] 47
#9 ((following or after) next concuss*):ti,ab,kw 56
#10 (#1 OR #2 OR #3 OR #4 OR #7 OR #8 OR #9) 661
#11 (infant* or toddler* or child* or boy* or girl* or infant* or juvenil* or minors or paediatric* or pediatric* or school* or preschool or pre-school* or kids or kindergarten or nursery or adolesc* or preadolesc* or pre-adolesc* or pubert* or pubescen* or prepube* or pre-pube* or high-school or highschool* or teen* or young or youth* or student* or undergrad* or college or campus):ti,ab,kw 357615
#12 (#10 and #11) 292
#13 ((infant* or toddler* or child* or boy* or girl* or infant* or juvenil* or minors or paediatric* or pediatric* or school* or preschool or pre-school* or kids or kindergarten or nursery or adolesc* or preadolesc* or pre-adolesc* or pubert* or pubescen* or prepube* or pre-pube* or high-school or highschool* or teen* or young or youth* or student* or undergrad* or college or campus or sport*) NEAR concuss*):ti,ab,kw 219
#14 (sport* next related concussion*):ti,ab,kw 85
#15 (#12 OR #13 OR #14) 402

****

Ovid APA PsycInfo *<1806 to March Week 2 2020>*

----

1 Post-Concussion Syndrome.mp. (510)
2 (((post or persistent or unresolved or delayed) adj4 concuss*) or postconcuss*).tw,id. (1769)
3 (commotio cerebri or post trauma* encephalopath* or posttrauma* encephalopath* or ((post commotion or postcommotion or post contusi* or postcontusi* or post head injur*) adj2 syndrom*) or (post trauma* syndrom* or posttrauma* syndrom*) or shell shock*).tw,id. (415)
4 (exp Brain Concussion/ or exp brain injuries/) and ((post or persistent or unresolved or delayed) adj4 (brain or skull or head or injur*)).ti,ab,id. (2597)
5 following concuss*.tw,id. (180)
6 (sport? adj2 concuss*).tw,id. (854)
7 or/1-6 (5055)
8 (adolescent or child).hw. (211965)
9 (infant? or toddler* or child* or boy* or girl* or juvenil* or minors or paediatric* or pediatric* or school* or preschool or pre-school* or kids or kindergarten or nursery or adolesc* or preadolesc* or pre-adolesc* or pubert* or pubescen* or prepube* or pre-pube* or high-school or highschool* or teen* or young or youth* or student* or undergrad* or college or campus).tw,id,hw. (1678306)
10 (infant* or child* or adolesc*).jw. (149631)
11 or/8-10 (1687636)
12 7 and 11 (1453)

****

***Ovid Embase** <1974 to 2020 Week 12>*

----

1 postconcussion syndrome/ (2154)
2 ((post or persistent or unresolved or delayed) adj4 concuss*).mp. or (postconcuss* or post concuss*).tw,kw. (4340)
3 (commotio cerebri or post trauma* encephalopath* or posttrauma* encephalopath*).mp. or ((post commotion or postcommotion or post contusi* or postcontusi* or post head injur*) adj2 syndrom*).tw,kw. or (post trauma* syndrom* or posttrauma* syndrom*).tw,kw. or shell shock*.tw,kw. (542)
4 exp concussion/ (11486)
5 ((post or persistent or unresolved or delayed) adj4 (brain or skull or head or injur*)).mp. (38875)
6 4 and 5 (972)
7 exp concussion/ and complication.fs. (989)
8 (following adj2 concuss*).mp. (1040)
9 or/1-3,6-8 (6911)
10 (infant* or adolescent or child* or pediatric* or paediatric*).jw,hw. (3431571)
11 (infant? or toddler* or child* or boy* or girl* or infant* or juvenil* or minors or paediatric* or pediatric* or school* or preschool or pre-school* or kids or kindergarten or nursery or adolesc* or preadolesc* or pre-adolesc* or pubert* or pubescen* or prepube* or pre-pube* or high-school or highschool* or teen* or young or youth* or student* or undergrad* or college or campus).ti,ab,kw. (3562249)
12 10 or 11 (4841571)
13 ((infant? or toddler* or child* or boy* or girl* or infant* or juvenil* or minors or paediatric* or pediatric* or school* or preschool or pre-school* or kindergarten or nursery or adolesc* or preadolesc* or pre-adolesc* or pubert* or pubescen* or prepube* or pre-pube* or high-school or highschool* or teen* or young or youth* or student* or undergrad*) adj3 concuss*).ti,kw. (879)
14 9 and 12 (2689)
15 13 or 14 (3110)
16 limit 15 to conference abstract status (878)
17 15 not 16 (2232)

****

Web of Science

Indexes=SCI-EXPANDED, SSCI Timespan=All years
#3 [2367] (#1 or #2)
#2 [851] TITLE: (((infant* or toddler* or child* or boy* or girl* or infant* or juvenil* or minors or paediatric* or pediatric* or school* or preschool or pre-school* or kindergarten or nursery or adolesc* or preadolesc* or pre-adolesc* or pubert* or pubescen* or prepube* or pre-pube* or high-school or highschool* or teen* or young or youth* or student* or undergrad*) NEAR/2 concuss*))
#1 [1801] TS=((((post or persistent or unresolved or delayed) NEAR concuss*) OR (postconcuss* or "post concuss*") OR ("commotio cerebri" or "post trauma* encephalopath*" or "posttrauma* encephalopath*") OR (("post commotion" or postcommotion or "post contusi*" or postcontusi* or "post head injur*") NEAR syndrom*) OR ("post trauma* syndrom*" or "posttrauma* syndrom*" OR "shell shock")) AND (infant* or toddler* or child* or boy* or girl* or infant* or juvenil* or minors or paediatric* or pediatric* or school* or preschool or pre-school* or kids or kindergarten or nursery or adolesc* or preadolesc* or pre-adolesc* or pubert* or pubescen* or prepube* or pre-pube* or high-school or highschool* or teen* or young or youth* or student* or undergrad* or college or campus))

****

EBSCO ERIC (Education Resources Information Center), BEI (British Education Index)
TI ((TBI or mTBI or “brain injur*” or "head injur*" or concuss* or postconcuss* or “post concuss*” or “commotio cerebri” or “post trauma* encephalopath*” or “posttrauma* encephalopath*” or “post commotion” or postcommotion or “post contusi*” or postcontusi* or “post head injur*” or “post trauma* syndrom*” or “posttrauma* syndrom*” or “shell shock”)) OR AB ((TBI or mTBI or “brain injur*” or "head injur*" or concuss* or postconcuss* or “post concuss*” or “commotio cerebri” or “post trauma* encephalopath*” or “posttrauma* encephalopath*” or“post commotion” or postcommotion or “post contusi*” or postcontusi* or “post head injur*” or “post trauma* syndrom*” or “posttrauma* syndrom*” or “shell shock”)) OR SU ((“Head Injuries” or "Brain Damage" or "Brain-Damaged Children” or "Concussion Policies”)) 1507

****

Search for Systematic Reviews-

Ovid MEDLINE(R) and Epub Ahead of Print, In-Process & Other Non-Indexed Citations and Daily <1946 to March 18, 2020>

----

1 ((((brain or head) adj1 trauma?) or ((brain injur* or encephalopath*) adj3 trauma*) or TBI? or MTBI? or sTBI?) adj3 (child* or boy* or girl* or juvenil* or minors or paediatric* or pediatric* or school* or kids or adolesc* or preadolesc* or pre-adolesc* or pubert* or pubescen* or prepube* or pre-pube* or teen* or young or youth* or student* or undergrad*)).ti,ab,kf. (3966)
2 *Brain Injuries/ or Brain Injuries, Traumatic/ or *Craniocerebral Trauma/ (62510)
3 adolescent/ or child/ (2770170)
4 adolescent.hw. (1999047)
5 (adolescent or child).hw. (3022224)
6 (child* or boy* or girl* or juvenil* or minors or paediatric* or pediatric* or school* or kids or adolesc* or preadolesc* or pre-adolesc* or pubert* or pubescen* or prepube* or pre-pube* or teen* or young or youth* or student* or undergrad*).ti,ab,kf. (2553130)
7 (child* or adolesc*).jw. (180288)
8 or/3-7 (4195274)
9 1 or (2 and 8) (20525)
10 (systematic or structured or evidence or trials or studies).ti. and ((review or overview or look or examination or update* or summary).ti. or review.pt.) (203502)
11 (0266-4623 or 1469-493X or 1366-5278 or 1530-440X or 2046-4053).is. (18549)
12 meta-analysis.pt. or (meta-analys* or meta analys* or metaanalys* or meta synth* or meta-synth* or metasynth*).ti,ab,kf,hw. (197549)
13 ((systematic or meta) adj2 (analys* or review)).ti,kf. or ((systematic* or quantitativ* or qualitativ* or methodologic*) adj5 (review* or overview*)).ti,ab,kf,sh. or ((quantitativ$ or qualitativ*) adj5 synthesis$).ti,ab,kf,hw. (266072)
14 (integrative research review* or research integration).tw. or scoping review?.ti,kf. or (review.ti,kf,pt. and (trials as topic or studies as topic).hw.) or (evidence adj3 review*).ti,ab,kf. (191477)
15 review.pt. and (medline or medlars or embase or pubmed or scisearch or psychinfo or psycinfo or psychlit or psyclit or cinahl or electronic database* or bibliographic database* or computeri#ed database* or online database* or pooling or pooled or mantel haenszel or peto or dersimonian or der simonian or fixed effect or ((hand adj2 search*) or (manual* adj2 search*))).tw,hw. (150616)
16 or/10-15 (551443)
17 9 and 16 (507)
18 (thematic adj (review or synthes* or summary)).mp. (895)
19 (mixed method? adj (synthes* or research or review)).mp. (1190)

20 ((integrative or realist) adj (synthes* or review)).mp. (3432)
21 (narrative adj (review or synthes* or summary)).mp. (12512)
22 evidence.ti. (227390)
23 or/18-22 (244469)
24 9 and 23 (153)
25 17 or 24 (605)

****

Eligibility criteria

We included observational studies that reported factors potentially associated with long-term adverse outcomes of mTBI. Using the World Health Organisation definition of childhood, we included studies reporting data on patients aged 0-18 years(14), and included any author-defined mTBI. Studies that assessed only children with clinically important TBI (ciTBI) with structural brain injury (15), or intentional/abusive TBI, were excluded. These injuries are often of greater severity with set management pathways, unlike unintentional mTBI which is expected to resolve spontaneously. Factors of interest were developed through consultation with clinicians who treat children with mTBI. These included demographics (e.g., socioeconomic status (SES), age, sex, ethnicity, parental education, family size, social services involvement, and any other parental or child characteristics), pre-morbid conditions (e.g., physical, sensory, behavioural, or learning difficulties prior to injury, or previous history of TBI), injury-related factors (e.g., mechanism, severity, Glasgow Coma Score (GCS), time and place of injury, and symptoms immediately post-injury), and biomarkers (blood biomarkers or imaging abnormalities). These factors were most relevant to the scope of our review, which aimed to create a list of factors to help clinicians pre-empt which children may need longer follow-up for PCS or other long-term adverse outcomes. We excluded genetic factors as these are hard to assess in emergency departments and difficult to modify, and treatment-related factors as pathways and options vary across healthcare systems and are better studied for effectiveness in intervention reviews.

Our primary outcome was presence of PCS three months or longer after mTBI. We included all PCS as defined by authors. We also included all author-defined recovery and other long-term adverse

outcomes of relevance to patients and clinicians (physical, behavioural, psychological) reported at 3 months or longer post-mTBI (16). We included all author-defined outcomes due to the absence of agreed definitions, so as not to miss any studied risk factors across all reported domains.

Study selection and data charting

We assessed abstracts and relevant full-text articles for eligibility in duplicate. One author extracted data from included studies and another checked a 20% sample. Extracted data included study design, author, location, year of publication, participants, risk factors identified or studied, and outcome(s) (Supplementary material).

As we aimed to comprehensively identify risk factors for adverse outcomes rather than quantify associations or assess their validity, we did not extract estimates (e.g., odds ratios) of their associations with outcomes. Methodological quality of included studies was not assessed for the same reason.

Synthesis

We extracted data for all demographic, premorbid and injury-related factors, or biomarker factors affecting primary and secondary outcomes on a standard template. Data were charted, thematically categorised and analysed iteratively after discussion with subject experts (JM, ML, IW). Findings were summarised into a table of risk factors studied for each outcome, and in figures presenting long-term adverse outcomes and risk factors studied in literature.

Stakeholder consultation

We presented preliminary findings to stakeholders comprising two young people with a history of PCS and their parents, and three clinicians who treat children with mTBI. This dialogue helped refine the synthesis and add patients' perspectives to our findings.

PRISMA Flow Diagram of inclusion process for the review

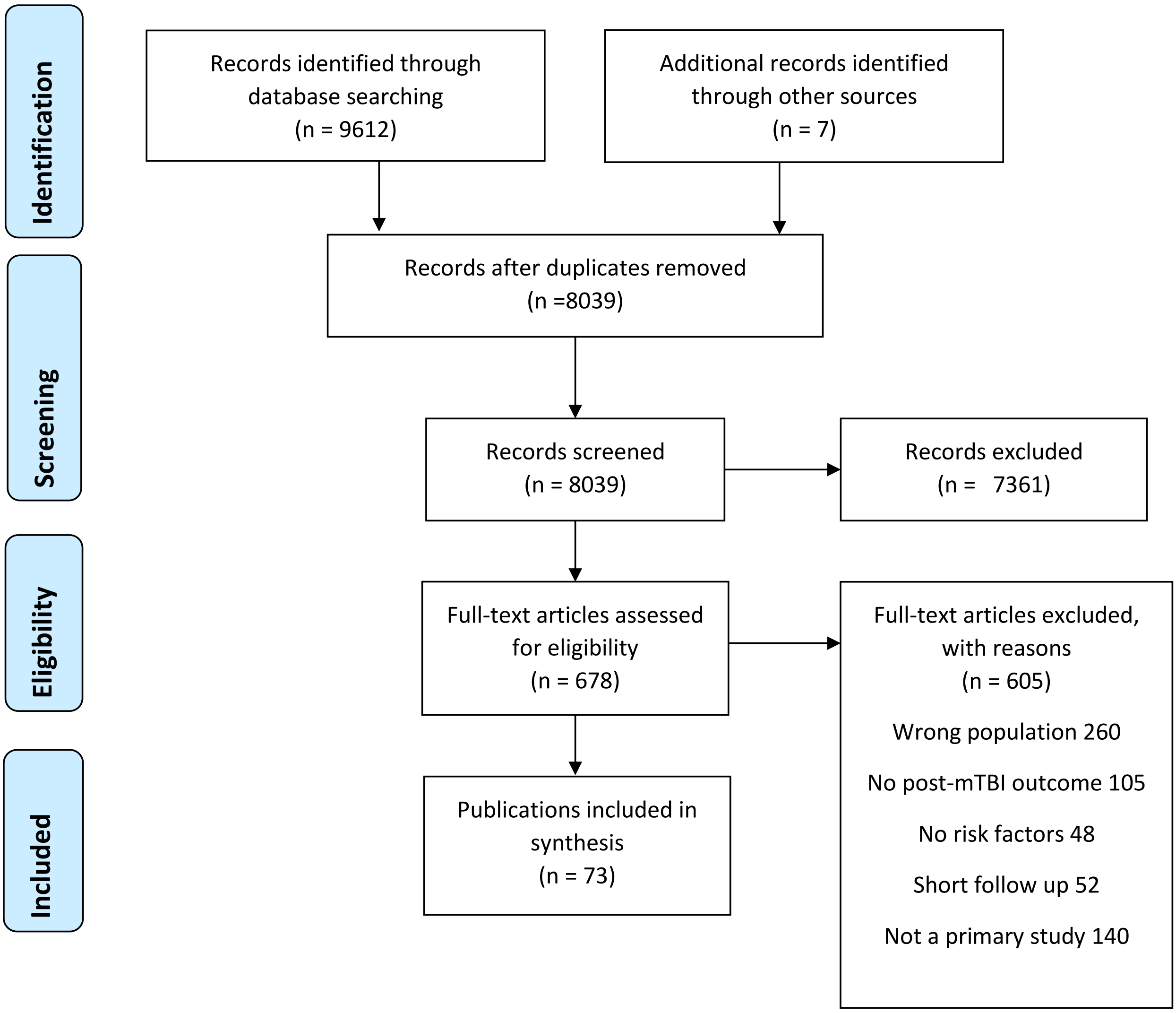

Supplementary Table of Risk factors and their relationship with each post-mTBI outcome in included studies

Outcomes<br>N studies	Risk Factors	Factors found to increase risk of adverse outcome<br>(N studies)	Factors found unrelated to outcome<br>(N studies)	Factors not reported in any included study for this outcome*
PCS<br>N=12	Demographic	Older age (1)<br>Being female (3)	Age (2)<br>Sex (1)<br>Ethnicity (1)<br>Sport played (1)<br>Insurance status (1)	SES, family function or rural/ urban residence, Family history of migraine or psychiatric illness, Intellect/ IQ of child, pubertal/ developmental status, Parental education.
	Premorbid	High parental distress (1)<br>Prior PCS symptoms (1)	Prior mTBI (1)<br>Child's IQ (1)<br>Premorbid anxiety (2)<br>Premorbid depression (2)<br>Prior ill health/medical conditions (1)	Prior headaches, pre-injury sleep problems, pre-injury ability/ disability, learning disorder, preinjury academic achievement, parental education, and family history of migraine.
	Injury related (immediate post injury)	Low psychological resilience (1)<br>Cognitive function (1)<br>High PCS symptoms (1)<br>Headache (1)<br>Needing a CT (1)	GCS scores (1)<br>Nausea/ vomiting (1)<br>Abnormalities on the CT (1)<br>5P risk score (1)<br>Loss of consciousness (1)<br>Amnesia (1)<br>Mode of arrival (1)<br>Mechanism of injury (2)<br>Time to presentation (1)<br>Time to diagnosis (1)	Fatigue, depression/ anxiety, sleep problems, cognition, memory. injury severity, location of event (injured at home, at field).
	Biomarkers	Serum S100B (1)	Abnormalities on CT (1)	Other serum biomarkers
Symptom score/severity<br>N=22	Demographic	Young age (1)<br>Being female (1)<br>Parental education (1)<br>SES (1)	Age (3)<br>Sex (5)<br>Family history of migraine or psychiatric illness (1)<br>Parental education (1)<br>SES (1)	Ethnicity, family function, sport played, or rural/ urban residence, Intellect/ IQ of child, pubertal/ developmental status,
	Premorbid	Prior mTBI (2)<br>Prior depression (1)<br>Prior anxiety (1)<br>ADHD (1)<br>Premorbid stress (1)	Prior mTBI (2)<br>Psychiatric diagnoses (3)<br>Premorbid anxiety (1)<br>ADHD (2)<br>Learning disability (1)<br>Preinjury migraine (2)<br>Sleep disorder (1)<br>Prior PCS- symptoms (2)<br>Prior physical disability (1)<br>School grades (1)	Pre-injury behaviour problems; Prior attention problems; prior distractibility; prior physical ill health; prior poor concentration
	Injury related	Sleep problems (2)<br>Low resilience (1)<br>High PTSD scores (1)<br>Injury mechanism (4)	Depression (1)<br>Amnesia (3)<br>Loss of consciousness (1)<br>injury mechanism (2)	5P risk score; cognition; concentration; fatigue; GCS score; headache; memory; nausea/vomiting; psychological resilience; injury severity; location of event (home, field); mode of arrival (EMT vs walk-in), injury severity, location of event (injured at home, at field).

Outcomes N studies	Risk Factors	Factors found to increase risk of adverse outcome (N studies)	Factors found unrelated to outcome (N studies)	Factors not reported in any included study for this outcome*
	Biomarkers	Serum marker S100B (1)	None reported	CT/MRI findings
Recovery N=29	Demographic	Older age (1)<br>Younger age (1)<br>Being female (6)<br>Being White (1)	Age (9)<br>Sex (5)<br>Ethnicity (4)<br>SES (2)<br>Rural/urban residence (1)<br>Sport played (1)<br>Insurance status (2)	Family function, Family history of migraine or psychiatric illness, Intellect/ IQ of child, pubertal/ developmental status, Parental education.
	Premorbid	Prior mTBI (4)<br>Premorbid headaches (3)<br>Prior PCS symptoms (1)<br>Pre-existing learning difficulties (1)<br>Lower school grades (1)<br>Headaches (3)<br>Preinjury sleep problems (1)	Prior mTBI (7)<br>Premorbid headaches (3)<br>Prior PCS symptoms (1)<br>Pre-existing learning difficulties (4)<br>Preinjury sleep problems (2)<br>Parental distress (3)<br>Prior comorbidities (1)<br>Prior physical disability (1)<br>Prior school grades (1)<br>Prior motion sickness (1)<br>Prior menstrual irregularities (1)	Pre-injury behaviour problems, Prior attention problems, prior distractibility, prior poor concentration, prior mental health
	Injury related	High 5P risk score (1)<br>High PCS symptoms (2)<br>Delayed diagnosis (1)<br>Low resilience (1)<br>Fatigue (1)<br>Depression (1)<br>Amnesia (1)<br>Poor cognition (1)<br>High internalizing symptoms (1)<br>Parental distress (3)<br>Loss of consciousness (2)<br>No Loss of consciousness (1)<br>Injury mechanism (3)<br>Higher severity of injury (1)<br>Continued activity participation (1)	GCS scores (1)<br>Anxiety (1)<br>Cognition (1)<br>Amnesia (1)<br>Loss of consciousness (3)<br>Injury mechanism (7)	Concentration, headache, memory, nausea/vomiting, psychological resilience, location of event (home, field), mode of arrival (EMT vs walk-in), time to presentation.
	Biomarker	None reported	None reported	CT/MRI findings, blood biomarkers
Cognitive function N=9	Demographic	Younger Age (1)<br>Being male (2)<br>Being female (1)<br>Low SES (2)<br>Being non-native Dutch (1)<br>Low parental education (1)<br>Rural residence (1)	Age (3)<br>Sex (1)<br>SES (2)<br>Low parental education (1)<br>Ethnicity (2)	Family function, family history of migraine or psychiatric illness, parental education, sport played

Ijaz S, *et al. Arch Dis Child* 2023; 108:492–497. doi: 10.1136/archdischild-2022-325202

Outcomes N studies	Risk Factors	Factors found to increase risk of adverse outcome (N studies)	Factors found unrelated to outcome (N studies)	Factors not reported in any included study for this outcome*
	Premorbid	ADHD (1)<br>Cognitive problems (1)	Developmental status-motor function of child (1)<br>Learning disorder (1)	Intellect/ IQ of child, pre-injury ability/ disability, pre-injury academic achievement, pre-injury behaviour problems, pre-injury PCS symptoms, pre-injury sleep problems, prior physical/mental ill health, prior headaches/migraine, prior poor concentration, prior mTBI.
	Injury related	Intentional mechanism (1)<br>Higher severity of injury (1)<br>No headache (1)	GCS scores (2)<br>PCS symptoms (2)<br>Loss of consciousness (2)<br>Amnesia (1)<br>Injury severity (1)<br>Injury mechanism (1)	5P risk score, cognition, depression/ anxiety, fatigue, memory, nausea /vomiting, psychological resilience, sleep problems, location of event (home, field), mode of arrival (EMT vs walk-in), time to presentation.
	Biomarker	Higher serum S100B (1)<br>MRI abnormalities (2)	MRI abnormalities (1)	CT findings, other blood biomarkers
Quality of Life N=9	Demographic	Younger age (2)<br>Being female (1)<br>High parental anxiety (1)<br>Low SES (1)	Age (2)<br>Sex (4)<br>Ethnicity (2)<br>SES (3)<br>Rural/urban residence (1)	Family function, Family history of migraine or psychiatric illness, parental education, Sport played,
	Premorbid	High parental distress (2)<br>Poor parent child interaction (1)	Prior mTBI (3)	Pre-injury ability/ disability, pre-injury academic achievement, Intellect/ IQ of child, pre-injury behaviour problems, pre-injury PCS symptoms, pre-injury sleep problems, Prior attention problems, prior distractibility, prior headaches/ migraine, prior learning problems, prior mental ill health, prior physical ill health, prior poor concentration, pubertal/ developmental status,
	Injury Related	High PCS symptoms (2)<br>Injury mechanism: Road accident (1)<br>Injury mechanism: intentional(1)	Cognitive function (1)<br>Injury mechanism (1)<br>Injury severity (2)	5P risk score, amnesia, biomarker levels, concentration, depression/ anxiety, fatigue, GCS score, headache, memory, Loss of consciousness, nausea/ vomiting, psychological resilience, sleep problems, location of event (home, field), mode of arrival (EMT vs walk-in), time to presentation
	Biomarker	None reported	Abnormalities on CT scans (1)	MRI findings, blood biomarkers
Behaviour N=6	Demographic	Low SES (1)<br>High parental anxiety (1)	Age (2)<br>Sex (2)<br>Ethnicity (2)<br>Rural urban residence (2)	Family function, family history of migraine or psychiatric illness, parental education, sport played.
	Premorbid	ADHD (1)	Prior mTBI (3)	Pre-injury ability/ disability, pre-injury academic achievement, Intellect/ IQ of child, pre-injury behaviour problems, pre-injury PCS symptoms, pre-injury sleep problems, prior distractibility, prior headaches / migraine, prior learning problems, prior physical/mental ill health, prior poor concentration, pubertal/ developmental status

Outcomes N studies	Risk Factors	Factors found to increase risk of adverse outcome (N studies)	Factors found unrelated to outcome (N studies)	Factors not reported in any included study for this outcome*
	Injury Related	Severe injury: complicated mTBI (1)<br>Motor vehicle injury mechanism (1)<br>No loss of consciousness (1)	Injury mechanism (2)	5P risk score, amnesia, biomarker levels, cognition, concentration, depression/ anxiety, fatigue, GCS score, headache, memory, nausea/ vomiting, psychological resilience, sleep problems, PCS-like symptoms location of event (home, field), mode of arrival (EMT vs walk-in), time to presentation
	Biomarker	MRI abnormalities (1)	MRI abnormalities (1)	Blood biomarkers, CT findings
Participation in activities N=3	Demographic	Poor family function (2)<br>Low SES (1)	Age (2)<br>Sex (2)<br>Ethnicity (1)	Family history of migraine or psychiatric illness, Parental anxiety /depression, Parental education, Rural/urban residence, Sport played
	Premorbid	Higher preinjury behaviour problems (1)	Developmental status of the child (1)<br>Prior comorbidities (1)	Intellect/ IQ of child, pre-injury ability/ disability, pre-injury academic achievement, pre-injury PCS symptoms, pre-injury sleep problems, Prior attention problems, prior mTBI, prior distractibility, prior headaches/ migraine, prior learning problems, prior mental ill health, Prior poor concentration
	Injury Related	High PCS symptoms (1)<br>High PTSD symptoms (1)	Fatigue (1)<br>GCS scores (1)<br>Amnesia (2)<br>Loss of consciousness (2) Injury severity (1)<br>Injury mechanism (1)	5P risk score, cognition, concentration, depression/ anxiety, headache, memory, nausea/vomiting, psychological resilience, sleep problems, location of event (home, field), mode of arrival (EMT vs walk-in), time to presentation
	Biomarker	None reported	None reported	Blood biomarkers, CT/MRI findings
Academic outcomes N=3	Demographic	High SES: private insurance (1)	Age (1)<br>Sex (1)<br>Ethnicity (1)<br>SES (1)	Family function, family history of migraine or psychiatric illness, Parental anxiety /depression, Parental education, Rural/urban residence, Sport played
	Premorbid	None reported	Prior mTBI (1)	Pre-injury ability/ disability, pre-injury academic achievement, Intellect/ IQ of child, pre-injury behaviour problems, pre-injury PCS symptoms, pre-injury sleep problems, Prior attention problems, prior distractibility, prior headaches/ migraine, prior learning problems, prior mental ill health, prior physical ill health, prior poor concentration, pubertal/ developmental status

Ijaz S, *et al. Arch Dis Child* 2023; 108:492–497. doi: 10.1136/archdischild-2022-325202

Outcomes N studies	Risk Factors	Factors found to increase risk of adverse outcome (N studies)	Factors found unrelated to outcome (N studies)	Factors not reported in any included study for this outcome*
	Injury Related	Higher PCS symptom (1)	None reported	5P risk score, amnesia, biomarker levels, cognition, concentration, depression/ anxiety, fatigue, GCS score, headache, memory, Loss of consciousness, nausea/ vomiting, psychological resilience, injury mechanism, injury severity sleep problems, location of event (home, field), mode of arrival (EMT vs walk-in), time to presentation
	Biomarker	None reported	None reported	Blood biomarkers, CT/MRI findings
Mental health N=7	Demographic	Sex (1)<br>Low SES (1)	Age (1)<br>Sex (2)	Ethnicity, family function or rural/ urban residence, family history of migraine or psychiatric illness, intellect/ IQ of child, pubertal/ developmental status, parental education, insurance status
	Premorbid	Prior mTBI (1)<br>Premorbid anxiety or depression (1)	Prior mTBI (1)	Pre-injury ability/ disability, pre-injury academic achievement, Intellect/ IQ of child, pre-injury behaviour problems, pre-injury PCS symptoms, pre-injury sleep problems, Prior attention problems, prior distractibility, prior headaches / migraine, prior learning problems, prior poor concentration, pubertal/ developmental status
	Injury related	Low resilience (1)<br>High PCS symptoms (2)	Mechanism of injury (1)<br>Loss of consciousness (1)	5P risk score, amnesia, cognition, concentration, depression/ anxiety, fatigue, GCS score, headache, memory, nausea/ vomiting, injury severity sleep problems, location of event (home, field), mode of arrival (EMT vs walk-in), time to presentation
	Biomarker	Weaker connectivity on MRI (1)		Blood biomarkers, CT findings
Headache N=4	Demographic	Younger age (1)<br>Being female (1)<br>Sport played: football, soccer or basketball (1)	None reported	SES, ethnicity, family function or rural/ urban residence, family history of migraine or psychiatric illness, intellect/ IQ of child, pubertal/ developmental status, parental education, insurance status
	Premorbid	Prior mTBI (3)<br>Loss of consciousness (1)<br>Prior headaches (1)	Prior headaches (1)	Pre-injury ability/ disability, pre-injury academic achievement, Intellect/ IQ of child, pre-injury behaviour problems, pre-injury PCS symptoms, pre-injury sleep problems, Prior attention problems, prior distractibility, prior headaches / migraine, prior learning problems, prior physical/mental ill health, Prior poor concentration, pubertal/ developmental status
	Injury related	Acute post injury headache (1)	Mechanism of injury (1)	PCS symptoms, 5P risk score, amnesia, biomarker levels, cognition, concentration, depression/ anxiety, fatigue, GCS score, headache, memory, Loss of consciousness, nausea/ vomiting, psychological resilience, Injury mechanism, injury severity sleep problems, location of event (home,

Ijaz S, *et al. Arch Dis Child* 2023; 108:492–497. doi: 10.1136/archdischild-2022-325202

Outcomes N studies	Risk Factors	Factors found to increase risk of adverse outcome (N studies)	Factors found unrelated to outcome (N studies)	Factors not reported in any included study for this outcome*
				field), mode of arrival (EMT vs walk-in), time to presentation
	Biomarker	None reported	None reported	CT/MRI findings, blood biomarkers
Vision N=1	Demographic	Young age (1)<br>Being female (1)	None reported	SES, ethnicity, family function or rural/ urban residence, family history of migraine or psychiatric illness, intellect/ IQ of child, pubertal/ developmental status, parental education, insurance status
	Premorbid	Learning difficulties/ ADHD (1)	None reported	Prior mTBI, pre-injury academic achievement, Intellect/ IQ of child, pre-injury behaviour problems, pre-injury PCS symptoms, pre-injury sleep problems, Prior attention problems, prior distractibility, prior headaches / migraine, prior learning problems, prior physical/mental ill health, prior poor concentration, pubertal/ developmental status
Sleep quality N=1		None reported	PSC symptoms (1)	No other factors assessed for this outcome

Table Legend: 5P: Predicting Persistent Post-Concussive Problems in Pediatrics; ADHD: Attention deficit hyperactivity disorder; CT: computerised tomography; EMT: emergency medical transport; GCS: Glasgow coma scale; IQ: intelligence quotient; MRI: magnetic resonance imaging; mTBI: mild traumatic brain injury; N= number; PCS: post-concussion syndrome; S100B: calcium-binding protein S100, beta isoform; SES: socioeconomic status.

*Where no study (N=0) reported an assessment of listed factor for this specific outcome

Table of Characteristic of Included studies

Study ID (author or pub.date)	Country	Year(s) of data collection	Sample Source/ Study Design	Type Of Follow Up	Injury types	Sample size (N analysed)	Age years at outcome assessment median (IQR) /mean± SD, (range)	Age years at injury	Gender % Females	Ethnicity % White	Follow up time(s) in months	Risk factors assessed	Outcomes assessed
**Aggarwal 2020 (1)**	USA	2012-2015	Patient record Review	Retrospective	Sport; non sport (RTA, fall, other)	227	15.73 ±1.49	.	25%	54	8	Sex; Ethnicity; prior concussion; prior ADHD; prior learning disability	Recovery
**Anaby 2012 (2)**	Canada	2001-2003	ED Cohort Followed Up	Prospective	RTA; others	136	11.5 ±3.5 (4-17)	.	35%	75%	8; 12	SES; Family function (FAD); injury severity; loss of consciousness; MOI	Participation frequency and diversity in activities
**Babcock 2013 (3)**	USA	2003 - 2004	ED Cohort Followed Up	Prospective	Fall; RTA	406	14 (5-18)	14	39%	84%	3	Age; sex; ethnicity; Insurance status; prior TBI; other prior diagnoses; headache at presentation; acute nausea/ vomiting; missed school days post injury; GCS; loss of consciousness, mode or arrival (EMS vs walk in); MOI; CT abnormal or not	PCS
**Babikian 2013 (4)**	USA	.	ED Cohort Followed Up	Prospective	.	76	12.7± 2	(8-17)	38%	.	12	Acute PCS symptoms; injury severity	Cognitive impairment
**Bateman, 2020 (5)**	.	2018-2019	Patient record Review	Retrospective	.	97	(11-20)	.	100%	.	5	Menstrual irregularities	Time to recovery
**Beauchamp 2018 (6)**	Canada	2013-2015	ED Cohort Followed Up	Prospective	.	196	11.7 (3.1)	(5-18)	35%	.	3	Age; sex; prior concussion; premorbid learning; disability; prior depression; acute PCS symptoms; loss of consciousness; acute amnesia	Cognitive inefficiency

Ijaz S, *et al. Arch Dis Child* 2023; 108:492–497. doi: 10.1136/archdischild-2022-325202

Study ID (author pub.date)	Country	Year (s) of data collection	Sample Source/ Study Design	Type Of Follow Up	Injury types	Sample size (N analysed)	Age years at outcome assessment median (IQR) /mean± SD, (range)	Age years at injury	Gender % Females	Ethnicity % White	Follow up time(s) in months	Risk factors assessed	Outcomes assessed
**Brooks 2016 (7)**	Canada	.	ED Cohort Followed Up	Prospective	Fall; blow to head	77	13.6 ± 2.6	(8-17)	41.60 %	90.90 %	1, 3	ED-based acute cognitive abilities	Recovery
**Bunt, 2021 (8)**	USA	2015-2020	Speciality Clinic/ Referred Case Series	Prospective	Sport	332	.	15 Median 15.1 Mean	46 %	78	3	Acute resilience within ten days of injury (score: low, average, high)	Recovery; post-concussion symptom severity; Anxiety; Depression
**Chendrasekhar, 2020 (9)**	USA	2015-2019	Patient record Review +Survey	Prospective	Fall; struck; other (Non sport)	100	.	(1-14)	66 %	.	4- 68	Age; MOI; injury severity (score)	PCS
**Choe 2012 (10)**	USA	.	Speciality Clinic/ Referred Case Series	Prospective	Sport; non sport	61	(0.5 - 21)	(0.5 - 21)	.	.	6	Premorbid headaches; MOI	Headache
**Chung2019 (11)**	USA	2015-2018	Outpatient Cohort	Prospective	Sport	517	14 (6-18)	(6-18)	46.40 %	61 %	3	Sleep quality after injury	Recovery; Time to recovery; post-concussion symptom severity
**Corwin 2020 (12)**	USA	2017-2019	ED Patient record Review	Retrospective	Sport; non-sport	244	median iqr 13 (10, 16)	.	49 %	44 %	3	Immediate (diagnosed at ED visit) versus delayed diagnosis (diagnosed at a follow-up visit)	PCS Time to recovery

**Study ID (author pub.date)**	**Country**	**Year (s) of data collection**	**Sample Source/ Study Design**	**Type Of Follow Up**	**Injury types**	**Sample size (N analysed)**	**Age years at outcome assessment median (IQR) /mean± SD, (range)**	**Age years at injury**	**Gender % Females**	**Ethnicity % White**	**Follow up time(s) in months**	**Risk factors assessed**	**Outcomes assessed**
**DeMatteo 2014 (13)**	Canada	2001-2003	ED Cohort Followed Up	Prospective	RTA; others traumatic; others non traumatic	59	9.4 (2.7)	(5-18)	41 %	.	60	Age; sex; any health problems prior to injury; injury severity; MOI	QoL
**Durish 2019 (14)**	Canada	.	Speciality Clinic/ Referred Case Series	Prospective	RTA; fall; Sport; blow to head	93	15.6(13-18)	.	56 %	92.40 %	5	Age; sex; acute Psychological Resilience (Connor-Davidson Resilience Scale (CD-RISC))	PCS
**Eisenberg 2013 (15)**	USA	2011-2012	ED Cohort Followed Up	Prospective	RTA; fall; sport; collision; blow to head	235	14.3	(11-22)	42.60 %	79 %	3	Age; sex; ethnicity; prior concussion; initial (acute) RPSQ score (PCS); prior migraine; prior ADHD; prior learning disability; MOI; acute loss of consciousness; acute amnesia; GCS	Time to recovery
**Fatima, 2020 (16)**	USA	2015-2020	Speciality Clinic/ Referred Case Series	Prospective	Sports	449	(12-18)	.	.	.	3	Acute PCS symptom score	Sleep quality
**Fehr 2017 (17)**	USA	2010-2012	Patient record Review of ED	Retrospective	RTA; fall; sport; other	431	14.3 ± 2.1	.	45.50 %	.	3	Age; sex; baseline/prior symptoms score; prior disability; prior ADHD; prior psychiatric diagnoses; prior concussion; prior migraine; prior sleep disorder; prior school grades; acute amnesia; loss of consciousness; MOI	Time to recovery; post-concussion symptom severity
**Gardner 2019 (18)**	USA	2010-2013	ED Patient record Review	Retrospective	.	285	10.6	(5-18)	36 %	.	>24	Age; sex; injury severity; prior concussion	Recovery; Time to recovery

Study ID (author or pub.date)	Country	Year(s) of data collection	Sample Source/ Study Design	Type Of Follow Up	Injury types	Sample size (N analysed)	Age years at outcome assessment median (IQR) /mean± SD, (range)	Age years at injury	Gender % Females	Ethnicity % White	Follow up time(s) in months	Risk factors assessed	Outcomes assessed
**Gravel 2020 (19)**	Canada	2013 - 2015	ED Cohort Followed Up	Prospective	RTA; sport; non sport; fall; assault; other	2240	12.3	(5-18)	.	.	3	Time to presentation	PCS
**Haase 2015 (20)**	USA	.	ED Patient record Review	Mix (Prospective + Retrospective)	.	141	(13-18)	.	53%	61%	6	Acute anxiety; acute depression	PCS; Time to recovery
**Hammer, 2021 (21)**	USA	2016-2018	Population Based Cohort Follow Up	Prospective	Sports	125	16.3±1.2	.	36%	.	3,6,12	Sex	Depression
**Heinzelmann, 2022 (22)**	USA	2015-2020	Speciality Clinic/ Referred Case Series	Prospective	Sport (football)	58	.	(10-24)	0%	.	3	MOI (playing surface: Astroturf vs grass)	Post-concussion symptom severity
**Heyer 2016 (23)**	USA	2012-2014	ED Patient record Review	Retrospective	Helmeted; not helmeted; other	1953	14.1 ± 2.1	(10-19)	36.60%	94.30%	≤ 12	Age; sex; ethnicity; insurance status; premorbid headaches; prior concussions; continued activity participation; symptoms on presentation day (acute PCS); MOI; amnesia; loss of consciousness	Recovery
**Howell 2018 (24)**	Canada	2013-2017	Patient record Review Of Referred Cases	Retrospective	.	230	14.8 ± 2.5	>18	50%	.	3 -12	5P risk score	PCS; Time to recovery

Study ID (author pub.date)	Country	Year (s) of data collection	Sample Source/ Study Design	Type Of Follow Up	Injury types	Sample size (N analysed)	Age years at outcome assessment median (IQR) /mean± SD, (range)	Age years at injury	Gender % Females	Ethnicity % White	Follow up time(s) in months	Risk factors assessed	Outcomes assessed
**Jeckell 2019 (25)**	USA	.	Speciality Clinic/ Referred Case Series	Prospective	Sport	136	15.58 ± 1.72 (12-20)	(12-20)	40 %	83.80 %	3	Sport played: Team vs. Individual sport	PCS
**Jones 2018(26)**	New Zealand	2010-2011	Follow Up Of Population Based Incident Cohort	Prospective	RTA; Fall; Blow to head; assault; unknown	222	8.36 ± 4.6 (2-15)	(2-15)	38 %	58 %	12	Age; sex; ethnicity; SES; rural/urban residence; parental anxiety post injury; prior TBI; MOI	Behaviour; Cognitive function; HRQoL
**Jones 2019 (27)**	New Zealand	2010-2011	Follow Up Of Population Based Incident Cohort	Prospective	RTA; Fall; Blow to head; assault; unknown	196	(1-15)	7.6 ± 4.8	37.20 %	59.20 %	48	Age; sex; SES; ethnicity; rural/urban residency; parental anxiety and depression; prior mTBI (concussion); MOI	Behaviour; Neurocognition ; HRQoL; Time to recovery
**Jones, 2021 (28)**	New Zealand	2010-2011	Population Based Cohort Follow Up	Prospective	RTA; fall; struck; other	93	Median 5	(0-11)	37.60 %	64.5	48	SES (semi/unskilled vs professional /skilled; Parental anxiety depression post injury	Psychosocial outcome
**Kamins, 2021(29)**	USA	2017-2019	Concussion Registry Data Review+ Follow Up	Prospective	Sport; non-sport	286	.	(5-18)	58.70 %	.	3,6	Acute headache (migraine vs non-migraine phenotype)	Recovery; Headache
**Kelmendi, 2021 (30)**	Kosovo	.	ED Cohort Follow Up	Prospective	RTA; sport; fall; other	60	11.1± 2.4	(7-16)	43.30 %	.	3	Serum S100B levels within 3 hr of injury	PCS

Ijaz S, *et al. Arch Dis Child* 2023; 108:492–497. doi: 10.1136/archdischild-2022-325202

**Study ID (author pub.date)**	**Country**	**Year(s) of data collection**	**Sample Source/ Study Design**	**Type Of Follow Up**	**Injury types**	**Sample size (N analysed)**	**Age years at outcome assessment median (IQR) /mean± SD, (range)**	**Age years at injury**	**Gender % Females**	**Ethnicity % White**	**Follow up time(s) in months**	**Risk factors assessed**	**Outcomes assessed**
**Lambregts 2018 (31)**	Netherlands	2008-2011	Repeat Cross-Sectional Survey	Prospective	.	73	12 (6-22)	.	43.80 %	.	24	Age; sex; ethnicity (Dutch or non-native Dutch); SES (parent education); preinjury developmental status of the child; GCS at admission; mTBI severity score; loss of consciousness; amnesia	Cognitive function; Participation in activities
**Ledoux 2019 (32)**	Canada	2013-2015	ED Cohort Followed Up	Prospective	Sport; non-sport; RTA; fall; assault; other	3063	Median (IQR) 12.0 (9.2-14.6)	(5-18)	39.30 %	.	3	Age; sex	Recovery Post concussion symptom severity
**Lumba-Brown, 2020 (33)**	Canada	2013-2015	ED Cohort Follow Up	Prospective	RTA; sport; non sport; assault	3029	12.9 (5-17)	(5-17)	39 %	.	3	MOI (RTA vs sport vs non sport vs assault)	Time to recovery; post-concussion symptom severity
**Maillard-Wermelinger 2009 (34)**	USA	.	ED Cohort Followed Up	Prospective	.	186	(8-15)	11.96 ± 2.2	29 %	71 %	3, 12	Loss of consciousness; presence of abnormalities on the MRI	Executive Function
**Mark 2012 (35)**	USA	.	ED Cohort Followed Up	Prospective	Sport; recreation; fall	186	(8-15)	.	29 %	73 %	3, 12	Prior ADHD; injury severity: complicated mild vs mild injury	Behaviour
**Martin, 2020 (36)**	USA	2018-2019	Patient record Review	Retrospective	RTA; Fall; sport; struck; other	637	Median (IQR): 13 (11–15)	(5-17)	53 %	.	3, 6, 12	Pre-existing anxiety disorder (Mental ill health)	Time to recovery; post-concussion

Study ID (author pub.date)	Country	Year (s) of data collection	Sample Source/ Study Design	Type Of Follow Up	Injury types	Sample size (N analysed)	Age years at outcome assessment median (IQR) /mean± SD, (range)	Age years at injury	Gender % Females	Ethnicity % White	Follow up time(s) in months	Risk factors assessed	Outcomes assessed
													symptom severity
**Martin, 2021 (37)**	USA	2018-2019	Patient record Review	Retrospective	RTA; fall; sport; struck; other	680	Median (IQR): 13 (11–15)	(5-17)	53 %	71 %	3, 6	Age; sex; prior learning disability; prior ADHD; MOI (RTA vs sport)	Time to recovery; post-concussion symptom severity; Vision symptoms at 3 months
**Nemade, 2022 (38)**	.	2013-2018	Patient record Review	Retrospective	Sport	603	13 ± 2.8 (5–18)	.	.	.	3, 6, 12	Age; sex; prior history of headaches; prior concussions; loss of consciousness; MOI (type of sport played)	Headache
**Olsson 2013 (39)**	Australia	.	ED Cohort Followed Up	Retrospective	RTA; sport; fall; blow to head	150	10.87 (6-16)	.	28 %	.	6, 18	Premorbid (prior) PCS; acute PCS; prior depression or anxiety score; HRQoL; Executive Function	PCS
**O'Neill 2019 (40)**	USA	.	Speciality Clinic/ Referred Case Series	Prospective	Sport; non sport; assault	42	15.2 ± 1.5 (13-18)	.	47.60 %	73.80 %	3	Age; sex; ethnicity; acute symptom severity score	Return to learn
**Painter 2017 (41)**	.	.	Speciality Clinic/ Referred Case Series	.	.	96	14.6 (6-18)	.	59 %	72 %	≤12	Prior ADHD/LD, migraines or psychiatric problems; prior anxiety and Depression; family history of migraines or psychiatric problems	Post-concussion symptom severity
**Papoutsis 2014 (42)**	Australia	2000-2001	ED Cohort Followed Up	Mix (Prospective And Retrospective)	.	52	(0-4)	1.5	56 %	.	84	Injury severity: mild vs complicated mild	Executive Function

Study ID (author pub.date)	Country	Year(s) of data collection	Sample Source/ Study Design	Type Of Follow Up	Injury types	Sample size (N analysed)	Age years at outcome assessment median (IQR) /mean± SD, (range)	Age years at injury	Gender % Females	Ethnicity % White	Follow up time(s) in months	Risk factors assessed	Outcomes assessed
**Parks 2010 (43)**	USA	.	ED Cohort Followed Up	Prospective	.	38	13.69 (6.22-18.84)	.	18 %	45 %	2, 12	MRI findings (lateral ventricular volume; third ventricular volume); GCS	Cognitive function
**Pate, 2022 (44)**	USA	2018 - 2019	Patient record Review	Retrospective	Sport; non sport	122	(5-18)	(5-18)	.	.	3	Age; MOI: sport vs non sport	Time to recovery
**Plourde 2018 (45)**	Canada	.	Follow Up of Cases Identified From Previous Studies	Prospective	.	75	14.3 (8-19)	.	52 %	64 %	32	Prior concussion	QoL; Behaviour
**Ponsford 1999 (46)**	Australia	.	ED Cohort Followed Up	Prospective	RTA; fall; sport;	130	(6-15)	.	30 %	.	3	Sex; prior TBI; prior psychological conditions; premorbid stressor (family breakdown)	Post-concussion Symptom severity
**Preiss-Farzanegan 2009 (47)**	USA	2003	Nested Cohort Follow Up	Mix (Prospective And Retrospective)	Sport	137	13.1 (4-17)	.	71 %	88 %	3	Sex	PCS; Post concussion symptoms severity
**Presley, 2020 (48)**	USA	.	Speciality Clinic/ Referred Case Series	Prospective	.	58	14.9 (13–18)	.	.	.	3	Premorbid psychiatric history (Mental ill health)	Post-concussion symptom severity
**Renaud 2019 (49)**	Netherlands	2015-2018	ED Cohort Followed Up	Prospective	RTA; sport	156	(6-18)	11.4 ± 3.3	34.60 %	.	6	Age; sex; SES; prior family functioning; preinjury (prior) function, behaviour; pre injury activity level; acute fatigue; acute PCS symptoms and PTSD symptoms; GCS; loss of consciousness; amnesia; MOI	Participation in activities

Study ID (author pub.date)	Country	Year (s) of data collection	Sample Source/ Study Design	Type Of Follow Up	Injury types	Sample size (N analysed)	Age years at outcome assessment median (IQR) /mean± SD, (range)	Age years at injury	Gender % Females	Ethnicity % White	Follow up time(s) in months	Risk factors assessed	Outcomes assessed
**Riemann, 2021 (50)**	Europe and Israel	2014-2017	Ed Cohort Follow Up	Prospective	.	196	Median (IQR) 17 (14–19)	(5-21)	28 %	.	6	Sex; CT abnormalities	PCS; HRQoL
**Rosenbaum, 2020 (51)**	USA	2017-2019	Concussion Registry Data Review+ Follow Up	Prospective	Sport; non sport	600	(5-18)	(5-18)	54.00 %	75.6	3	Age; sex; prior number of comorbidities; prior concussion; migraine history (headache); prior anxiety and/or depression (Mental ill health)	Time to recovery
**Stazyk 2017 (52)**	Canada	2013	ED Cohort Followed Up	Prospective	RTA; sport; fall; other	92	15 ± 2.5 (7-18)	.	60.80 %	.	3.5 - 35	Sex; prior concussion; acute PCSI score	Depression
**Stein 2017 (53)**	USA	2014-2015	Speciality Clinic/ Referred Case Series	Prospective	Sport; recreational activities	49	15 ± 1.63 (11-17)	.	65.30 %	75.50 %	3, 6	Acute PCS score; acute depression score	Post-concussion symptom severity
**Studer 2014 (54)**	Switzerland	2012	ED Cohort Followed Up	Prospective	RTA, Fall; blow to head; other	40	(6-16)	11 ± 3.1	56.40 %	.	4	Preinjury attention problems	Post-concussion symptoms severity; Neuropsychological function; Socio-behavioural symptoms
**Studer**	Switzerl	2012-	ED Cohort Followed Up	Prospective	.	36	(6-16)	11 ±	55 %	.	4	Acute serum marker S100B	Post-concussion

Ijaz S, *et al. Arch Dis Child* 2023; 108:492–497. doi: 10.1136/archdischild-2022-325202

Study ID (author pub.date)	Country	Year(s) of data collection	Sample Source/ Study Design	Type Of Follow Up	Injury types	Sample size (N analysed)	Age years at outcome assessment median (IQR) /mean± SD, (range)	Age years at injury	Gender % Females	Ethnicity % White	Follow up time(s) in months	Risk factors assessed	Outcomes assessed
**2015(55)**	and	2013						3.07					symptom severity
**Sufrinko 2017 (56)**	USA	2014	Speciality Clinic/ Referred Case Series	Prospective	Sport	69	15.3± 1.9	(12-22)	26%	.	3	Acute visual motor speed; cognitive-migraine-fatigue symptoms within the first 7 days (acute)	Recovery
**Tanenbaum 2018 (57)**	USA	2009-2011	Sport Cohort Follow Up	Prospective	Sport	26	15.6 ± 1.02 (7-17)	.	15%	.	4	Preinjury (prior) ADHD; learning disability	Cognitive inefficiency
**Tarkenton, 2020 (58)**	USA	.	Speciality Clinic/ Referred Case Series	Prospective	RTA; fall; sport; hit	440	(12-18)	.	.	.	3	Sex; PTSD Checklist (PCL-5) score at initial visit; prior depression; prior anxiety; MOI (unclear, likely MVA vs others); acute post traumatic amnesia	Post-concussion symptom severity
**Tarkenton, 2021 (59)**	USA	2015-2021	Speciality Clinic/ Referred Case Series	Prospective	.	70	15.4 ± 1.8	.	69%	74	3	MOI (RTA vs sport)	Recovery; post-concussion symptom severity
**Taylor 2015 (60)**	USA	2001-2006	ED Cohort Followed Up	Prospective	RTA; fall; sport; other	176	(8-15)	11.94 ± 2.23	30%	71%	3, 12	Loss of consciousness, MOI, MRI abnormalities	Behaviour
**Teel, 2021 (61)**	Canada	.	.	Prospective	Sport; other	49	12	.	55%	.	3	Acute parental stress; premorbid parental anxiety;	Time to recovery;
**Tham 2019 (62)**	USA	2016	ED Cohort Followed Up	Prospective	Sport; fall; blow to head	29	13.96 ± 1.83(11-18)	(11-18)	69%	79.30%	3	Sleep quality after injury (acute)	Post-concussion symptom severity

Ijaz S, et al. Arch Dis Child 2023; 108:492–497. doi: 10.1136/archdischild-2022-325202

Study ID (author or pub.date)	Country	Year(s) of data collection	Sample Source/ Study Design	Type Of Follow Up	Injury types	Sample size (N analysed)	Age years at outcome assessment median (IQR) /mean± SD, (range)	Age years at injury	Gender % Females	Ethnicity % White	Follow up time(s) in months	Risk factors assessed	Outcomes assessed
**Truss 2017 (63)**	Australia	2013-2015	ED Cohort Followed Up	Prospective	RTA; sport; fall; other	120	12 ±2.4 (8-18)	.	25 %	.	3	Age; sex; premorbid anxiety or depression; acute PTSD symptomatology: resilient vs recovering or chronic; loss of consciousness; MOI	PTSD
**Truss, 2021 (64)**	Australia	2013-2015	Ed Cohort Follow Up	Prospective	RTA; Fall; sports	169	11.7± 3.2	.	30 %	.	3	Age; sex; prior concussion; pre-injury PCS (PCSI total score); premorbid learning disorder; premorbid headaches; premorbid motion sickness; premorbid sleep problems; internalising and externalising symptoms on CBCL T; premorbid parental distress; MOI; loss of consciousness; CT done or not	Recovery
**Tuerk, 2020 (65)**	Canada	2011-2015	ED Cohort Follow Up	Prospective	.	52	(1.5 - 5)	.	39 %	98	6, 18	Age; sex; SES; parent child interaction; prior parental distress; acute PCS symptoms; acute cognition; injury severity	QoL
**van Iersse l, 2021 (66)**	Canada	.	ED Cohort Follow Up	Prospective	RTA; sport; non sport; assault	432	11.5	.	38 %	.	14	Prior concussion	Recovery; post-concussion symptom severity; Headache; Return to sport; School functioning
**Vaughn, 2021 (67)**	USA	2011-2016	ED Cohort Follow Up	Prospective	RTA	50	12.33 ± 2.17	.	0.51	95	7	MRI connectivity at 7 weeks post mTBI	Post-concussion symptom severity; Anxiety
**Worrall, 2022 (68)**	USA	2015-2020	Not reported	Prospective	Sports	1197	(12-18)	.	.	.	3	Prior concussion	Recovery; post-concussion symptom severity;

Ijaz S, *et al. Arch Dis Child* 2023; 108:492–497. doi: 10.1136/archdischild-2022-325202

Study ID (author pub.date)	Country	Year (s) of data collection	Sample Source/ Study Design	Type Of Follow Up	Injury types	Sample size (N analysed)	Age years at outcome assessment median (IQR) /mean± SD, (range)	Age years at injury	Gender % Females	Ethnicity % White	Follow up time(s) in months	Risk factors assessed	Outcomes assessed
													Anxiety; Depression
**Wright, 2021 (69)**	USA	.	Outpatient Patient record Review+ Questionnaire Follow Up	Prospective	RTA; fall; sports; collision; assault	298	15.1± 1.8	.	52 %	80	3	Age; sex; prior concussion; loss of consciousness	Recovery
**Yeates 2012 (70)**	USA	.	ED Cohort Followed Up	Prospective	.	186	(8-15)	11.96 ± 2.2	29 %	71 %	3, 12	Acute high PCS symptoms (post TBI)	HRQoL
**Zonfrillo, 2021 (71)**	USA	2013-2015	ED Cohort Follow Up	Prospective	All	123	13.2± 3	8 to 18	28.50 %	79.7	6,12, 24	SES; Caregiver/parent education	HRQoL; post-concussion symptom severity; Executive Function
**Zuckerman 2016 (72)**	USA	2012-2015	Patient record Review+ Interviews	Retrospective	Sport	180	15.9 ± 2.0	.	30 %	84 %	12	Type of sport played: soccer, football, basketball etc MOI: contact mechanism (ground, body etc), player mechanism (defence vs shooting kick off etc); aware or unaware of oncoming collision	Time to recovery
**Zuckerman 2017 (73)**	USA	2012-2015	Patient record Review+ Interviews	Retrospective	Sport	282	15.8 (11.6-22.2)	.	38.60 %	85.50 %	12	SES	Time to recovery; Days of missed school; Days of missed practice

5 P = Predicting and Preventing Post-concussive Problems in Pediatrics, ADHD= attention deficit hyperactivity disorder, CT= computerized tomography, ED= emergency department, EMS= emergency medical services, FAD= Family assessment device, GCS= Glasgow coma scale, HRQol= Health related quality of life, IQR= interquartile range, LD= learning disability, MOI= mechanism of injury, MRI= magnetic resonance imaging, MVA=motor vehicle accident, N=number, PCL5= Posttraumatic Checklist for DSM-5, PCS= post-concussion syndrome, PCSI= post-concussion symptom inventory, PTSD= post-traumatic stress disorder, Qol= quality of life, RTA= road traffic accident, RPSQ=

Rivermead post-concussion symptom questionnaire, S100B= calcium-binding protein S100, beta isoform SES= socioeconomic status, SD= standard deviation, TBI= traumatic brain injury,

1. Aggarwal SS, Ott SD, Padhye NS, Schulz PE. Sex, race, ADHD, and prior concussions as predictors of concussion recovery in adolescents. 2020;**34**:809-17 Online.
2. Anaby D, Law M, Hanna S, DeMatteo C. Predictors of Change in Participation Rates Following Acquired Brain Injury: Results of a Longitudinal Study. *Developmental Medicine & Child Neurology*. 2012;**54**:339-46 Online.
3. Babcock L, Byczkowski T, Wade SL, Ho M, Mookerjee S, Bazarian JJ. Predicting postconcussion syndrome after mild traumatic brain injury in children and adolescents who present to the emergency department. *JAMA pediatrics*. 2013;**167**:156-61 Online.
4. Babikian T, McArthur D, Asarnow RF. Predictors of 1-month and 1-year neurocognitive functioning from the UCLA longitudinal mild, uncomplicated, pediatric traumatic brain injury study. *Journal of the International Neuropsychological Society*. 2013;**19**:145-54 Online.
5. Bateman E, Streeter L, Mortazavi M, Minor J, Handmaker H. Incidence and Patterns of Menstrual Irregularities in Pediatric Patients After Concussion. *Neurology*. 2020;**95**:S16-S Online.
6. Beauchamp MH, Aglipay M, Yeates KO*, et al.* Predictors of neuropsychological outcome after pediatric concussion. *Neuropsychology*. 2018;**32**:495-508 Online.
7. Brooks BL, Daya H, Khan S, Carlson HL, Mikrogianakis A, Barlow KM. Cognition in the Emergency Department as a Predictor of Recovery after Pediatric Mild Traumatic Brain Injury. *Journal of the International Neuropsychological Society*. 2016;**22**:379-87 Online.
8. Bunt SC, Meredith-Duliba T, Didehhani N*, et al.* Resilience and recovery from sports related concussion in adolescents and young adults. 2021:1-12 Online.
9. Chendrasekhar A, Kuczabski B, Cohen D*, et al.* Delayed Sequelae Related to Mild Traumatic Brain Injury in Children. *Global Pediatric Health*. 2020;**7** Online.
10. Choe M, Valino H, McArthur D, Giza C. Risk Factors for Development of Post-Concussive Headaches in a Pediatric TBI Subspecialty Clinic Population. *Neurology*. 2012;**78**:2 Online.
11. Chung JS, Zynda AJ, Didehbani N*, et al.* Association Between Sleep Quality and Recovery Following Sport-Related Concussion in Pediatrics. *Journal of Child Neurology*. 2019;**34**:639-45 Online.
12. Corwin DJ, Arbogast KB, Haber RA*, et al.* Characteristics and Outcomes for Delayed Diagnosis of Concussion in Pediatric Patients Presenting to the Emergency Department. *J Emerg Med*. 2020;**59**:795-804 Online.

13. DeMatteo CA, Hanna SE, Yousefi-Nooraie R*, et al.* Quality-of-life after brain injury in childhood: Time, not severity, is the significant factor. *Brain injury*. 2014;**28**:114-21 Online.
14. Durish CL, Yeates KO, Brooks BL. Psychological Resilience as a Predictor of Symptom Severity in Adolescents With Poor Recovery Following Concussion. *Journal of the International Neuropsychological Society*. 2019;**25**:346-54 Online.
15. Eisenberg MA, Andrea J, Meehan W, Mannix R. Time interval between concussions and symptom duration. *Pediatrics*. 2013;**132**:8-17 Online.
16. Fatima H, Caze T, Meredith-Duliba T*, et al.* An Effect of Pre-injury Factors on the Relationship between Severity of Acute Symptoms and Sleep Quality Among Recovered and Non-Recovered Adolescents following Concussion. *Archives of Clinical Neuropsychology*. 2020;**35**:922- Online.
17. Fehr SD, Nelson LD, Scharer KR*, et al.* Risk Factors for Prolonged Symptoms of Mild Traumatic Brain Injury: A Pediatric Sports Concussion Clinic Cohort. *Clinical Journal of Sport Medicine*. 2019;**29**:11-7 Online.
18. Gardner JE, Teramoto M, Hansen C. Factors Associated With Degree and Length of Recovery in Children With Mild and Complicated Mild Traumatic Brain Injury. *Neurosurgery*. 2019;**85**:E842-E50 Online.
19. Gravel J, Ledoux AA, Tang K*, et al.* Early versus delayed emergency department presentation following mild Traumatic Brain Injury and the presence of symptom at 1, 4 and 12 weeks in children. *Emergency Medicine Journal*. 2020;**5**:05 Online.
20. Haase E. Impacts of depression and anxiety on an adolescent's concussion recovery: An examination of mediation and moderation effects. *Dissertation Abstracts International: Section B: The Sciences and Engineering*. 2015;**76**:No-Specified Online.
21. Hammer E, Hetzel S, Pfaller A, McGuine T. Longitudinal Assessment of Depressive Symptoms After Sport-Related Concussion in a Cohort of High School Athletes. *Sports health*. 2021;**13**:31-6 Online.
22. Heinzelmann MM, Stokes M, Bunt S, Didehbani N, Miller S, Cullum M. Impact of Playing Surface on Concussion Symptoms in Young American Football Players. *Neurology*. 2022;**98**:S12 Online.
23. Heyer GL, Schaffer CE, Rose SC, Young JA, McNally KA, Fischer AN. Specific Factors Influence Postconcussion Symptom Duration among Youth Referred to a Sports Concussion Clinic. *Journal of Pediatrics*. 2016;**174**:33-8.e2 Online.
24. Howell DR, Zemek R, Brilliant AN, Mannix RC, Master CL, Meehan WP, 3rd. Identifying Persistent Postconcussion Symptom Risk in a Pediatric Sports Medicine Clinic. *The American journal of sports medicine*. 2018;**46**:3254-61 doi: 10.1177/0363546518796830 [published Online First: 2018/09/29].
25. Jeckell AS, Brett BL, Totten DJ, Solomon GS. Team versus individual sport participation as a modifying factor in the development of post-concussion syndrome after first concussion: A pilot study. *Applied Neuropsychology Child*. 2019;**8**:199-205 Online.
26. Jones KM, Barker-Collo S, Parmar P*, et al.* Trajectories in health recovery in the 12 months following a mild traumatic brain injury in children: findings from the BIONIC Study. *Journal of Primary Health Care*. 2018;**10**:81-9 Online.
27. Jones KM, Prah P, Starkey N*, et al.* Longitudinal patterns of behavior, cognition, and quality of life after mild traumatic brain injury in children: BIONIC study findings. *Brain injury*. 2019;**33**:884-93 Online.
28. Jones KM, Ameratunga S, Starkey NJ*, et al.* Psychosocial functioning at 4-years after pediatric mild traumatic brain injury. *Brain injury*. 2021;**35**:416-25 Online.
29. Kamins J, Richards R, Barney BJ*, et al.* Evaluation of Posttraumatic Headache Phenotype and Recovery Time After Youth Concussion. *JAMA Network Open*. 2021;**4**:e211312 Online.

30. Kelmendi FM, Morina AA, Mekaj AY*, et al.* Ability of S100B to predict post-concussion syndrome in paediatric patients who present to the emergency department with mild traumatic brain injury. 2021:1-6 Online.
31. Lambregts SAM, Smetsers JEM, Verhoeven IMAJ*, et al.* Cognitive function and participation in children and youth with mild traumatic brain injury two years after injury. *Brain injury*. 2018;**32**:230-41 Online.
32. Ledoux AA, Tang K, Yeates KO*, et al.* Natural Progression of Symptom Change and Recovery From Concussion in a Pediatric Population. *JAMA pediatrics*. 2019;**173**:e183820 Online.
33. Lumba-Brown A, Tang K, Yeates KO, Zemek R, Pediatric Emergency Research Canada PCT. Post-concussion symptom burden in children following motor vehicle collisions. 2020;**1**:938-46 Online.
34. Maillard-Wermelinger A, Yeates KO, Taylor HG*, et al.* Mild traumatic brain injury and executive functions in school-aged children. *Developmental Neurorehabilitation*. 2009;**12**:330-41 Online.
35. Mark EM. The influence of premorbid attention and behavior problems on neurobehavioral outcomes from pediatric mild traumatic brain injury. *Dissertation Abstracts International: Section B: The Sciences and Engineering*. 2012;**73**:624 Online.
36. Martin AK, Petersen AJ, Sesma HW*, et al.* Concussion symptomology and recovery in children and adolescents with pre-existing anxiety. *Journal of Neurology, Neurosurgery & Psychiatry*. 2020;**91**:1060-6 Online.
37. Martin AK, Petersen AJ, Sesma HW*, et al.* Learning and Attention Deficit/Hyperactivity Disorders as Risk Factors for Prolonged Concussion Recovery in Children and Adolescents. *Journal of the International Neuropsychological Society*. 2021:1-14 Online.
38. Nemade DP, Cottrill N, Payne M. Prevalence and Duration of Post-Concussive Headaches in a Pediatric Sports Clinic: A Cross-sectional Study. 2022;**98**:S7 Online.
39. Olsson KA, Lloyd OT, Lebrocque RM, McKinlay L, Anderson VA, Kenardy JA. Predictors of child post-concussion symptoms at 6 and 18 months following mild traumatic brain injury. *Brain injury*. 2013;**27**:145-57 Online.
40. O'Neill JA. Neuropsychological functioning and return to learn among adolescents with concussion. *Dissertation Abstracts International: Section B: The Sciences and Engineering*. 2019;**80**:No-Specified Online.
41. Painter K, Dodd J. Is There a Cumulative Effect of Risk Factors on Pediatric Postconcussive Syndrome? *Archives of Clinical Neuropsychology*. 2017;**32**:719- Online.
42. Papoutsis J, Stargatt R, Catroppa C. Long-term executive functioning outcomes for complicated and uncomplicated mild traumatic brain injury sustained in early childhood. *Developmental Neuropsychology*. 2014;**39**:638-45 Online.
43. Parks LK. Longitudinal changes in ventricle volume following pediatric traumatic brain injury: Predictors of cognitive function one year later. *Dissertation Abstracts International: Section B: The Sciences and Engineering*. 2010;**70**:5179 Online.
44. Pate J, Cummins I, Cooper K, Mooney J, Chandler McLeod M, Gould S. Evaluation of Sports-Related Concussion in Children (5-12) and Adolescents (13-18). *Neurology*. 2022;**98**:S12-S3 Online.
45. Plourde V, Yeates KO, Brooks BL. Predictors of Long-Term Psychosocial Functioning and Health-Related Quality of Life in Children and Adolescents With Prior Concussions. *Journal of the International Neuropsychological Society*. 2018;**24**:540-8 Online.

46. Ponsford J, Willmott C, Rothwell A*, et al.* Cognitive and behavioral outcome following mild traumatic head injury in children. *Journal of Head Trauma Rehabilitation*. 1999;**14**:360-72 Online.
47. Preiss-Farzanegan SJ, Chapman B, Wong TM, Wu J, Bazarian JJ. The relationship between gender and postconcussion symptoms after sport-related mild traumatic brain injury. *Pm & R*. 2009;**1**:245-53 Online.
48. Presley C, Caze T, Tarkenton T*, et al.* Post-Concussive Symptoms in Adolescent Athletes with Premorbid Psychiatric History: A Matched Case-Control Study. *Archives of Clinical Neuropsychology*. 2020;**35**:941- Online.
49. Renaud MI, Lambregts SAM, van de Port IGL, Catsman-Berrevoets CE, van Heugten CM. Predictors of activities and participation six months after mild traumatic brain injury in children and adolescents. *European Journal of Paediatric Neurology*. 2019;**3**:03 Online.
50. Riemann L, Voormolen DC, Rauen K, Zweckberger K, Unterberg A, Younsi A. Persistent postconcussive symptoms in children and adolescents with mild traumatic brain injury receiving initial head computed tomography. *Journal of Neurosurgery: Pediatrics*. 2021;**27**:538-47 Online.
51. Rosenbaum PE, Locandro C, Chrisman SPD*, et al.* Characteristics of Pediatric Mild Traumatic Brain Injury and Recovery in a Concussion Clinic Population. *JAMA Network Open*. 2020;**3**:e2021463 Online.
52. Stazyk K, DeMatteo C, Moll S, Missiuna C. Depression in youth recovering from concussion: Correlates and predictors. *Brain injury*. 2017;**31**:631-8 Online.
53. Stein E, Howard W, Rowhani-Rahbar A, Rivara FP, Zatzick D, McCarty CA. Longitudinal trajectories of post-concussive and depressive symptoms in adolescents with prolonged recovery from concussion. *Brain injury*. 2017;**31**:1736-44 Online.
54. Studer M, Goeggel Simonetti B, Joeris A*, et al.* Post-concussive symptoms and neuropsychological performance in the post-acute period following pediatric mild traumatic brain injury. *Journal of the International Neuropsychological Society*. 2014;**20**:982-93 Online.
55. Studer M, Goeggel Simonetti B, Heinks T*, et al.* Acute S100B in serum is associated with cognitive symptoms and memory performance 4 months after paediatric mild traumatic brain injury. *Brain injury*. 2015;**29**:1667-73 Online.
56. Sufrinko AM, Marchetti GF, Cohen PE, Elbin RJ, Re V, Kontos AP. Using Acute Performance on a Comprehensive Neurocognitive, Vestibular, and Ocular Motor Assessment Battery to Predict Recovery Duration After Sport-Related Concussions. *American Journal of Sports Medicine*. 2017;**45**:1187-94 Online.
57. Tanenbaum JB. Individual and collective effects of Attention Deficit/Hyperactivity Disorder and Learning Disabilities on changes in neurocognitive functioning in adolescents and young adults following a sports-related concussion. *Dissertation Abstracts International: Section B: The Sciences and Engineering*. 2018;**78**:No-Specified Online.
58. Tarkenton T, Presley C, Meredith-Duliba T*, et al.* The Contribution of Psychological Stress to Adolescent Post-Concussive Symptoms Across Recovery. *Archives of Clinical Neuropsychology*. 2020;**35**:784- Online.
59. Tarkenton T, Caze Ii T, Silver CH*, et al.* Differences in Adolescent Symptom Reporting Following Motor Vehicle Accident Versus Sport-Related Concussion. 2021;**36**:554-60 Online.
60. Taylor HG, Orchinik LJ, Minich N*, et al.* Symptoms of Persistent Behavior Problems in Children With Mild Traumatic Brain Injury. *Journal of Head Trauma Rehabilitation*. 2015;**30**:302-10 Online.

61. Teel EF, Caron JG, Gagnon IJ. Higher parental stress is significantly related to longer clinical recovery times in concussed children: A mixed-methods study. *Journal of Science & Medicine in Sport*. 2021;**20**:20 Online.
62. Tham SW, Aaron RV, Palermo TM. The role of sleep deficiency in the trajectory of postconcussive symptoms in adolescents. *Brain injury*. 2019;**33**:1413-9 Online.
63. Truss K, Godfrey C, Takagi M*, et al.* Trajectories and Risk Factors for Post-Traumatic Stress Symptoms following Pediatric Concussion. *Journal of neurotrauma*. 2017;**34**:2272-9 Online.
64. Truss K, Hearps SJC, Babl FE*, et al.* Trajectories and risk factors for pediatric postconcussive symptom recovery. *Neurosurgery*. 2021;**88**:36-45 Online.
65. Tuerk C, Gagner C, Degeilh F*, et al.* Quality of life 6 and 18 months after mild traumatic brain injury in early childhood: An exploratory study of the role of genetic, environmental, injury, and child factors. *Brain Res*. 2020;**1748**:147061 Online.
66. van Ierssel J, Ledoux AA, Tang K*, et al.* Symptom Burden, School Function, and Physical Activity One Year Following Pediatric Concussion. *Journal of Pediatrics*. 2021;**228**:190-8.e3 Online.
67. Vaughn KA, DeMaster D, Kook JH, Vannucci M, Ewing-Cobbs L. Effective connectivity in the default mode network after paediatric traumatic brain injury. *Eur J Neurosci*. 2021;**29**:29 Online.
68. Worrall H, Chung J, Cullum M, Miller S. Specialist Referral and Outcomes in Adolescent Athletes With Prior Concussion History. *Neurology*. 2022;**98**:S19-S20 Online.
69. Wright B, Wilmoth K, Juengst SB, Didehbani N, Maize R, Cullum CM. Perceived Recovery and Self-Reported Functioning in Adolescents with Mild Traumatic Brain Injury: The Role of Sleep, Mood, and Physical Symptoms. *Developmental neurorehabilitation*. 2021;**24**:237-43 Online.
70. Yeates KO, Kaizar E, Rusin J*, et al.* Reliable change in postconcussive symptoms and its functional consequences among children with mild traumatic brain injury. *Archives of Pediatrics & Adolescent Medicine*. 2012;**166**:615-22 Online.
71. Zonfrillo MR, Haarbauer-Krupa J, Wang J*, et al.* Effect of parental education and household poverty on recovery after traumatic brain injury in school-aged children. *Brain injury*. 2021;**35**:1371-81 Online.
72. Zuckerman SL, Totten DJ, Rubel KE, Kuhn AW, Yengo-Kahn AM, Solomon GS. Mechanisms of Injury as a Diagnostic Predictor of Sport-Related Concussion Severity in Football, Basketball, and Soccer: Results From a Regional Concussion Registry. *Neurosurgery*. 2016;**63**:102-12 Online.
73. Zuckerman SL, Zalneraitis BH, Totten DJ*, et al.* Socioeconomic status and outcomes after sport-related concussion: a preliminary investigation. *Journal of Neurosurgery Pediatrics*. 2017;**19**:652-61 Online.

Ijaz S, *et al. Arch Dis Child* 2023; 108:492–497. doi: 10.1136/archdischild-2022-325202
